# Supplementary material for: FAM111A protects replication forks from protein obstacles via its trypsin-like domain
Source: Nat Commun. 2020 Mar 12;11:1318. doi: 10.1038/s41467-020-15170-7 (PMC7067828; doi:10.1038/s41467-020-15170-7)
Supplement: Supplementary file 1 — Supplementary Information [file 41467_2020_15170_MOESM1_ESM.pdf]

Supplementary Information

FAM111A Protects Replication Forks from Protein Obstacles via Its Trypsin-like Domain

Kojima et al.

Supplementary Figures

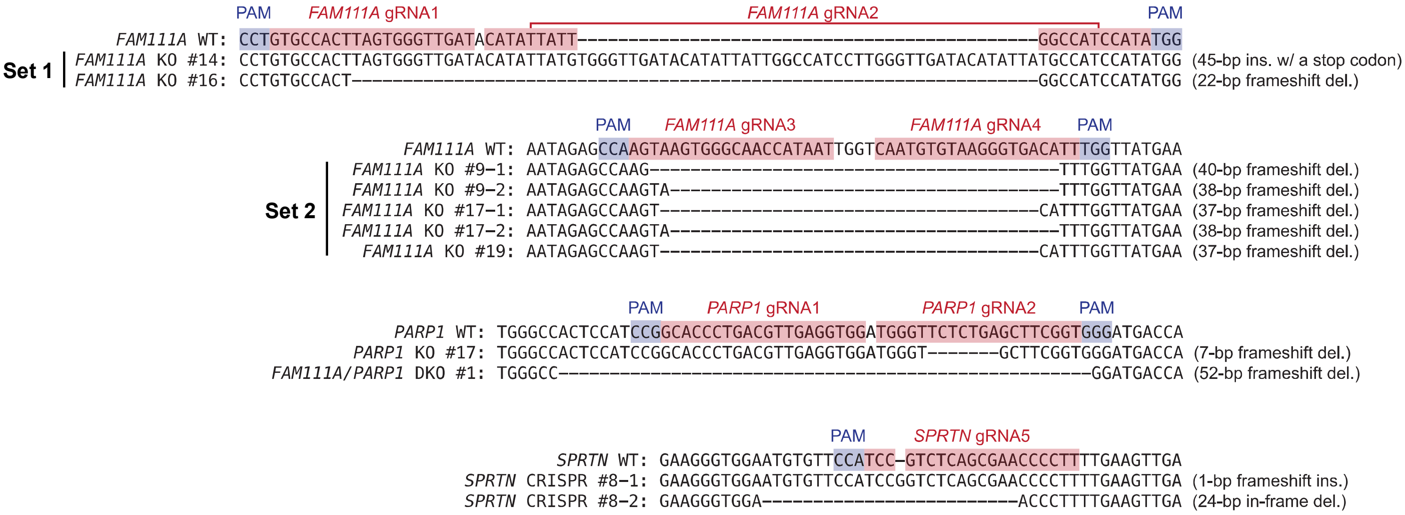

Supplementary Figure 1. Summary of mutations generated by CRISPR/Cas9. Sequencing results of HAP1 clones used in this study are shown. Target sequences of gRNAs and the PAM sequences are indicated in red and blue, respectively. *FAM111A/PARP1*-DKO clone #1 is a derivative of *FAM111A*-KO clone #14 (set 1 gRNA).

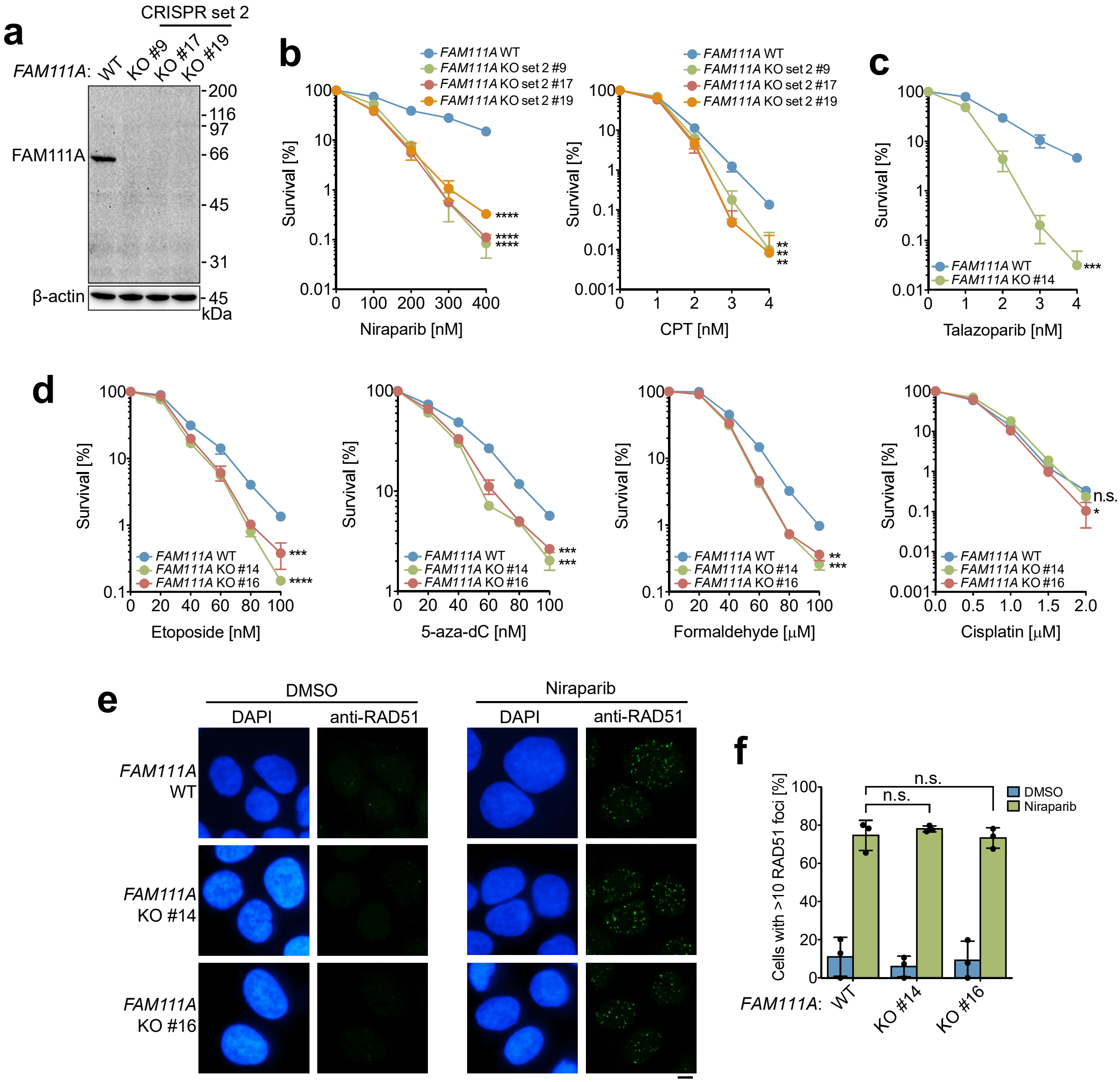

Supplementary Figure 2. Sensitivities of *FAM111A* KO cells to various agents. (a) Knockout of *FAM111A* by CRISPR/-Cas9 (set 2 gRNA) in HAP1. *FAM111A*-KO HAP1 clones (KO #9, #17 and #19) were generated using set 2 gRNAs and the indicated proteins were analyzed by Western blotting. **(b)** Clonogenic survival assays. Cells analyzed in (a) were cultured in the presence of the indicated concentration of niraparib or CPT for 6 days. **(c,d)** Clonogenic survival assays. Parental HAP1 (WT) and *FAM111A*-KO clones analyzed in Fig. 1a were cultured in the presence of the indicated concentrations of talazoparib (c) , etoposide (d), 5-aza-dC (d) or cisplatin (d) for 6 days. For formaldehyde (d), cells were treated for the first 24 hr and cultured for the remaining 5 days without the chemical. In (b), (c) and (d), results shown are representative of three independent experiments and values are mean ± s.d. of technical replicates (n = 3). p-values were calculated relative to *FAM111A* WT. ****p<0.0001; ***p<0.001; **p<0.01; *p<0.05; n.s., not significant (two-tailed unpaired t-test). **(e)** Detection of RAD51 foci after niraparib treatment. Parental HAP1 (WT) or *FAM111A*-KO clones (#14 and #16) were treated with 2 μM niraparib or DMSO for 5 hr and stained with anti-RAD51 antibody and DAPI. Scale bar, 5 μm. **(f)** Quantification of cells containing RAD51 foci. Experiments were performed as in (e). At least 100 cells were scored by an investigator blinded to sample identity and the percentages of cells with 10 or more foci are shown. Values are mean ± s.d. of independent experiments (n = 3). n.s., not significant (two-tailed unpaired t-test). Source data are provided as a Source Data file.

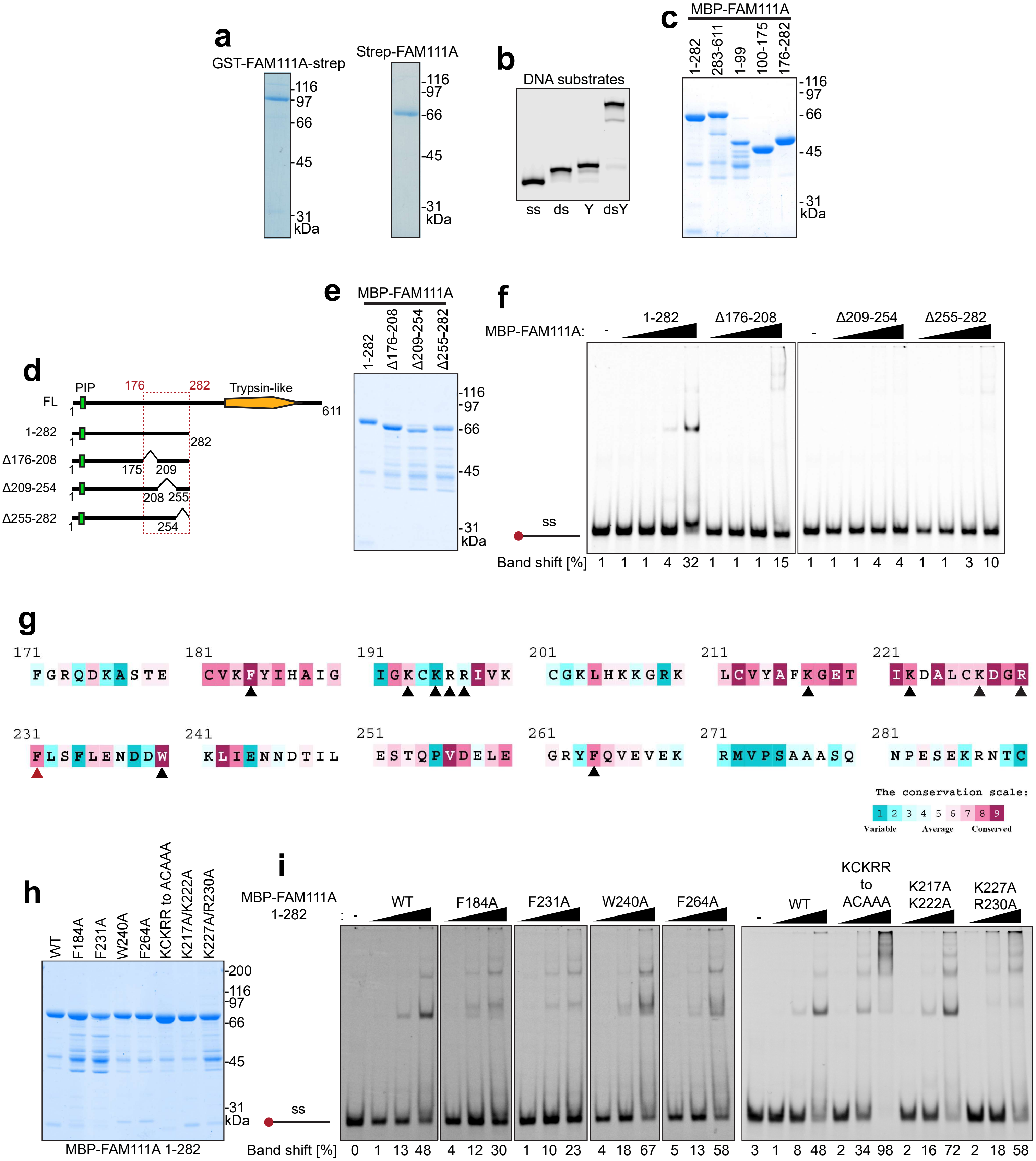

Supplementary Figure 3

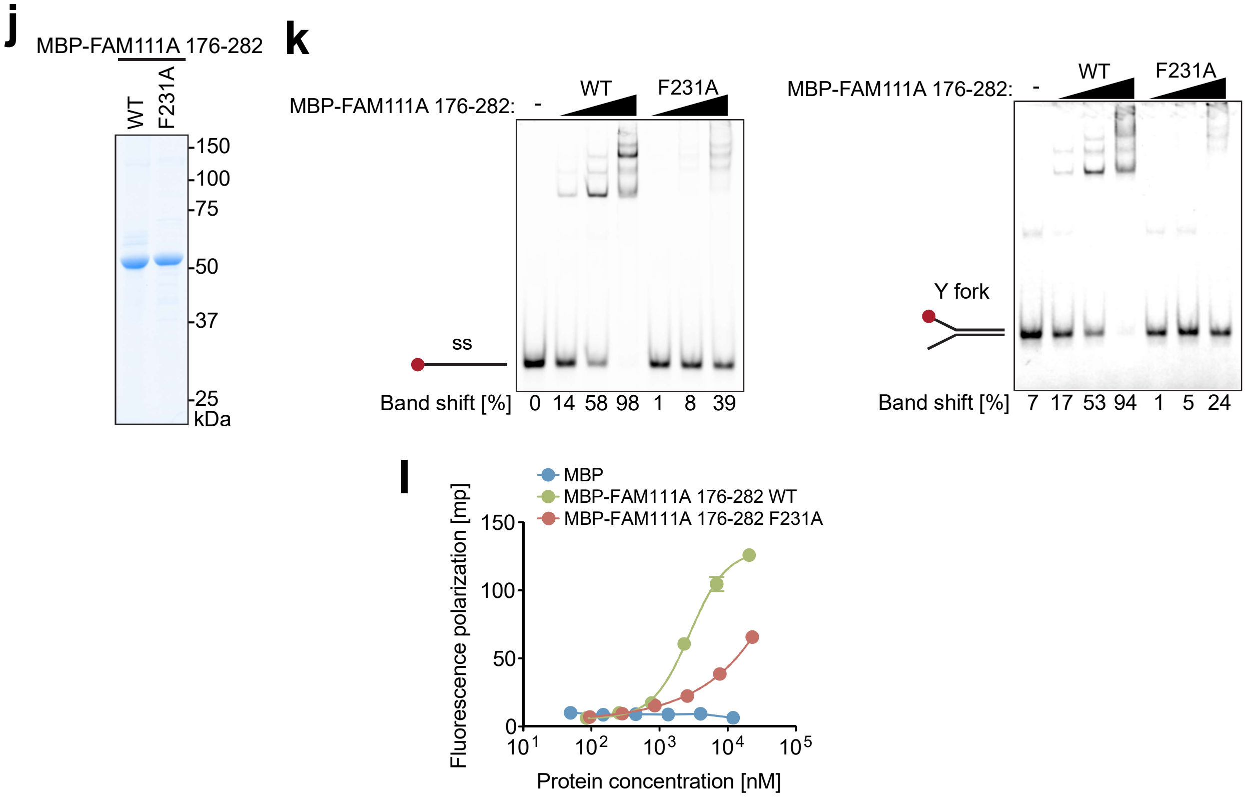

Supplementary Figure 3. FAM111A binds to ssDNA through its central region (a) Purified recombinant FAM111A proteins. GST-FAM111A-strep (left) used in Fig. 2a,b and Strep-FAM111A (right) used in Fig. 2c are shown. **(b)** DNA substrates used for EMSA. IRDye700-labeled DNA substrates (ss: single-stranded, ds: double-stranded, Y: Y-fork, dsY: double-stranded Y-fork) analyzed by polyacrylamide gel electrophoresis are shown. **(c)** Purified recombinant MBP-FAM111A proteins used in Fig. 2e. **(d)** Schematic representation of deletions within the FAM111A central region. **(e)** Purified MBP-FAM111A 1-282 with a deletion indicated in (d). **(f)** EMSA using MBP-FAM111A 1-282 analyzed in (e). Increasing amounts of the indicated MBP-FAM111A 1-282 (50, 150, 450, and 1350 nM) were incubated with IRDye700-labeled ssDNA oligos. MBP (1350 nM) was added in the "-" lane. *, nonspecific band. **(g)** The amino acid sequence of FAM111A central region. Residues mutated in this study are indicated by arrowheads (a red arrowhead indicates Phe231 that was mutated (F231A) and used for further analyses). Degree of amino acid conservation among species was analyzed by the ConSurf server and indicated by the color scale shown below. **(h)** Purified MBP-FAM111A 1-282 proteins with point mutations. **(i)** EMSA using MBP-FAM111A 1-282. Increasing amounts of the indicated MBP-FAM111A 1-282 species (50, 150, 450, and 1350 nM for WT and equivalent amounts for mutants) were incubated with IRDye700-labeled ssDNA oligo nucleotides. MBP was added in the "-" lane as a control. **(j)** Purified MBP-FAM111A 176-282 WT or F231A proteins (**k**) EMSA using MBP-FAM111A 176-282. Increasing amounts of the indicated MBP-FAM111A 176-282 WT or F231A protein (50, 150, 450, and 1350 nM) were incubated with IRDye700-labeled ssDNA (left) or Y-fork (right) oligo nucleotides. MBP was added in the "-" lane as a control. (**l**) FP DNA binding assays. Various amounts of recombinant MBP-FAM111A 176-282 proteins (WT or F231A) or MBP were mixed with 6-FAM-labeled ssDNA and FP values were measured. Values are mean ± s.d. of independent experiments (n = 3). In (f), (i) and (k), percentages of band shifts in each lane are shown below. Red dots indicate IRDye700. Source data are provided as a Source Data file.

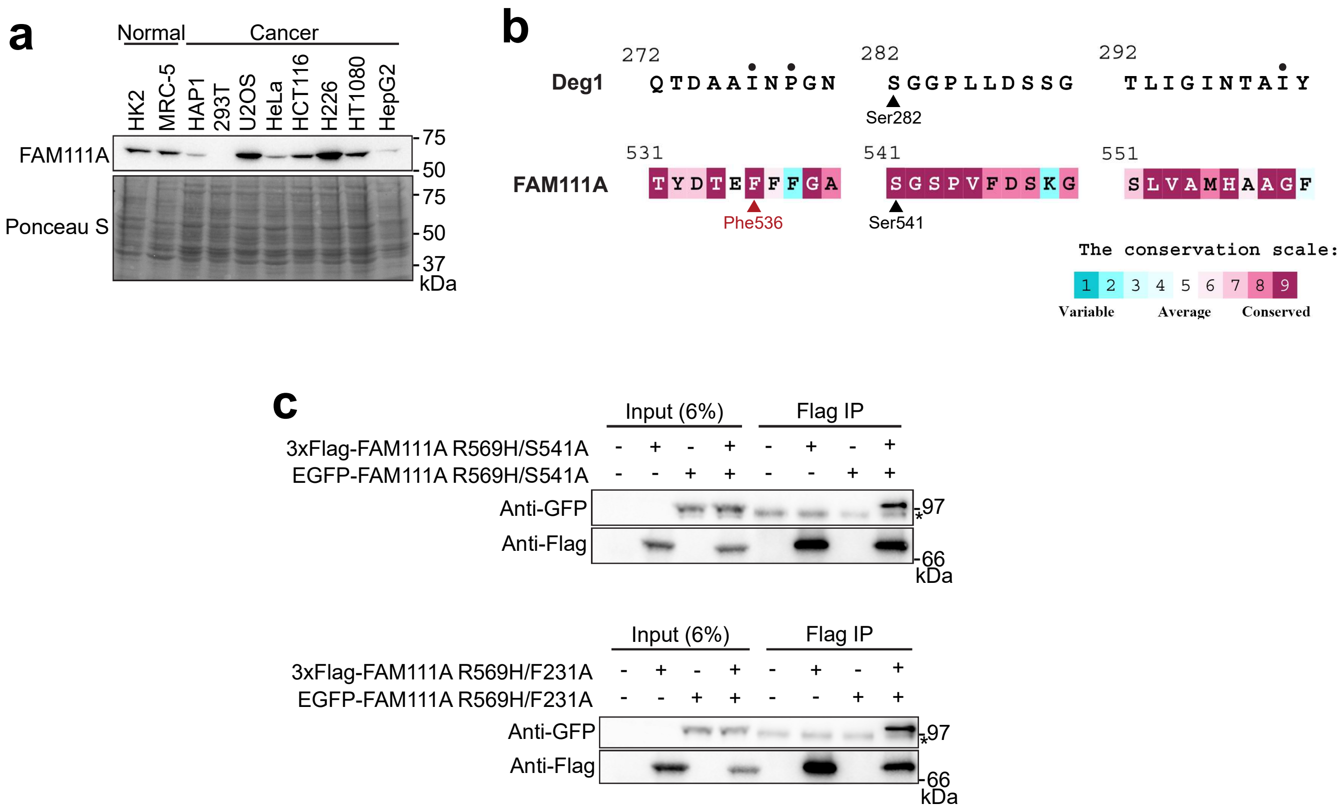

Supplementary Figure 4. FAM111A levels in various cell lines, and *in vivo* autocleavage and oligomerization of FAM111A. (a) Endogenous expression levels of FAM111A in different cell lines. Endogenous FAM111A proteins in the indicated cell lines were analyzed by Western blotting. Ponceau S-stained proteins are shown as loading control. **(b)** Alignment of amino acid sequences around the S1 pocket between *Arabidopsis thaliana* Deg1 (upper) and *Homo sapiens* FAM111A (lower). The black dots indicate the amino acid residues that consist the S1 pocket of Deg1, and the red arrowhead denotes FAM111A Phe536, a notable hydrophobic feature in the predicted FAM111A S1 pocket. The black arrowheads indicate active site serine residues for each protein. Degree of amino acid conservation among species was analyzed by the ConSurf server and indicated by the color scale shown below. **(c)** Co-immunoprecipitation assays. 3xFlag- and EGFP-FAM111A muants (upper pannel: R569H/S541A, lower pannel: R569H/F231A) were transiently expressed in 293T cells as indicated and anti-Flag immunoprecipitation was performed. Input and precipitated proteins were analyzed by Western blotting using the indicated antibodies. Empty vectors were used in the “-“ lanes. *, nonspecific band.

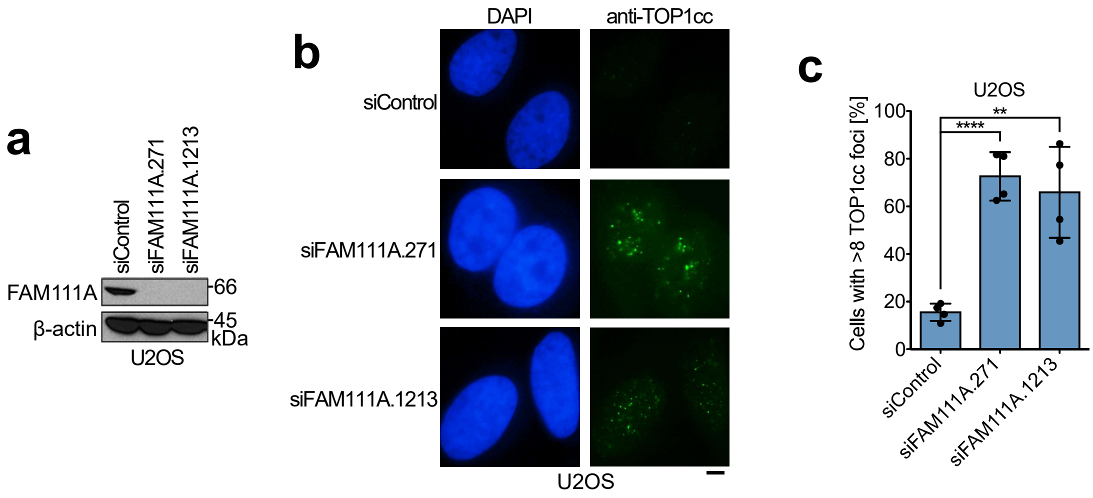

Supplementary Figure 5. TOP1cc accumulation in U2OS cells caused by knockdown of FAM111A. (a) Knockdown of FAM111A by RNAi. U2OS cells were transfected with siRNA oligos and the indicated proteins were examined by Western blotting. **(b)** TOP1cc focus formation. Cells analyzed in (a) were stained with anti-TOP1cc antibody and DAPI. Scale bar, 5 μm. **(c)** Quantification of cells containing TOP1cc foci. Experiments were performed as in (b). At least 100 cells were scored by an investigator blinded to sample identity and the percentages of cells with 8 or more foci recorded. Values are mean ± s.d. of independent experiments (n = 4). ****p<0.0001; **p<0.01 (two-tailed unpaired t-test). Source data are provided as a Source Data file.

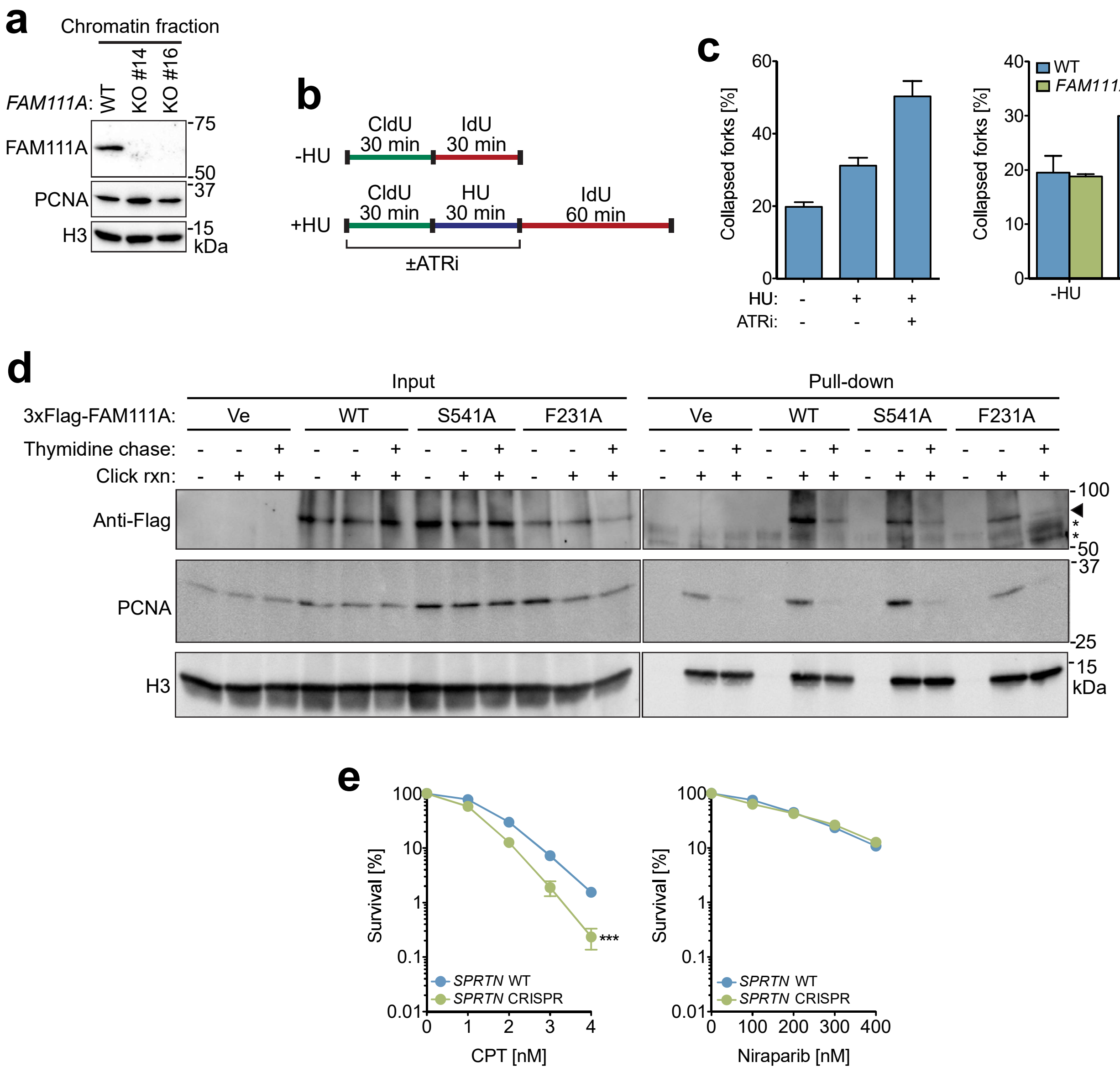

Supplementary Figure 6. Fffects of *FAM111A* KO on replication forks. (a) Detection of proteins on chromatin. Indicated proteins in the chromatin fration were detected by Western blotting. **(b)** Schematic representation of DNA combing assays for measuring collapsed forks. Cells were pulse-labeled with CldU for 30 min, treated with 5 mM HU for 30 min and released into IdU-containing media for 60 min. Where indicated, 5 µM ATR inhibitor (ATRi) was added during CldU labeling and HU treatment. In the experiments without HU treatment, cells were sequentially labeled with CldU and IdU for 30 min each. **(c)** Analyses of replication fork restart. Experiments were performed as in (a) and quantification of collapsed replication forks are shown as percentages of labeled tracts containing only CldU in all labeled tracts containing CldU. The left panel shows a positive control experiment in HAP1, where ATRi (ATR inhibitor, VE-821) augmented the percentage of collapsed replication forks induced by HU. In the right panel, parental HAP1 (WT) and *FAM111A* KO #14 were treated with or without HU and the percentages of collapsed forks were quantified. At least 200 labeled tracts in each sample were scored by an investigator blinded to sample identity. Values are mean ± s.d. of independent experiments (n = 2). **(d)** iPOND assays. 293T cells stably expressing 3xFlag-FAM111A (WT or indicated mutants) were lablelled with BrdU for 20 min with or without thymidine chase for 30 min. Pulled down proteins were detected by Western blotting. Ve: empty vector. The arrow head indicates 3xFlag-FAM111A. *: non-specific band. **(e)** Clonogenic survival assays. Parental HAP1 (WT) and *SPRTN* CRISPR #8 were cultured in the presence of the indicated concentration of CPT or niraparib for 6 days. Results shown are representative of two independent experiments and values are mean ± s.d. of technical replicates (n = 3). ***p<0.001 (two-tailed unpaired t-test). Source data are provided as a Source Data file.

Supplementary Figure 7. Uncropped blots.

Fig. 1a

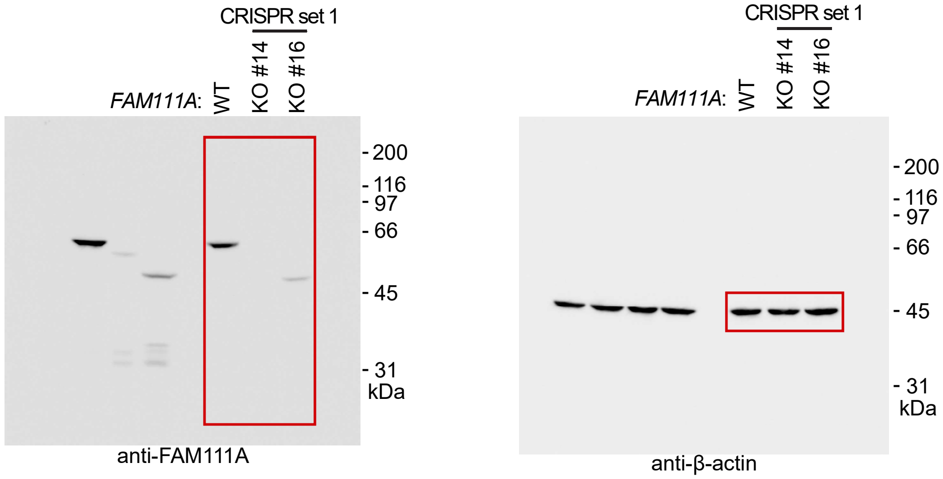

Fig. 2a

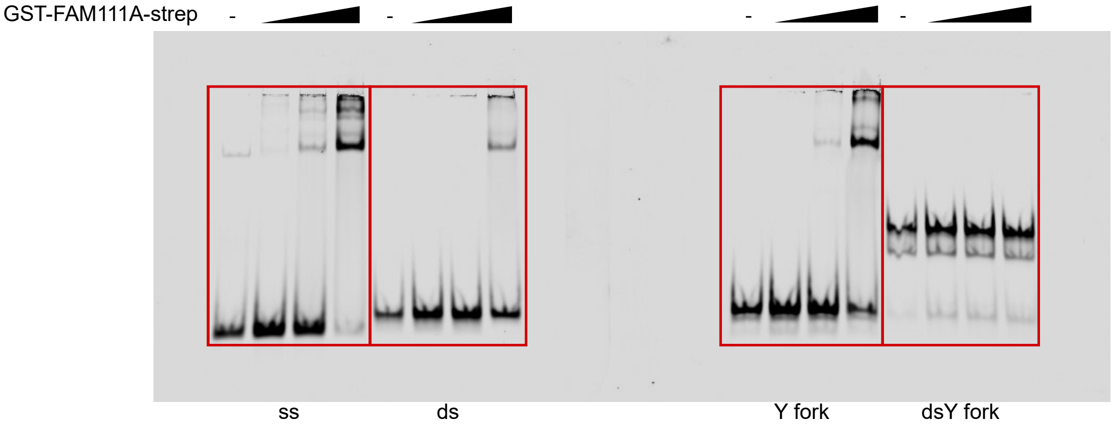

Fig. 2b

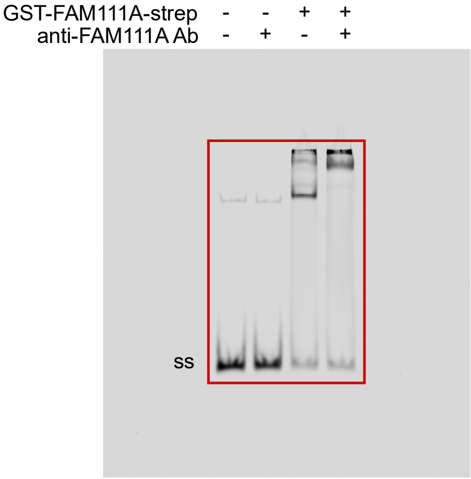

Fig. 2e

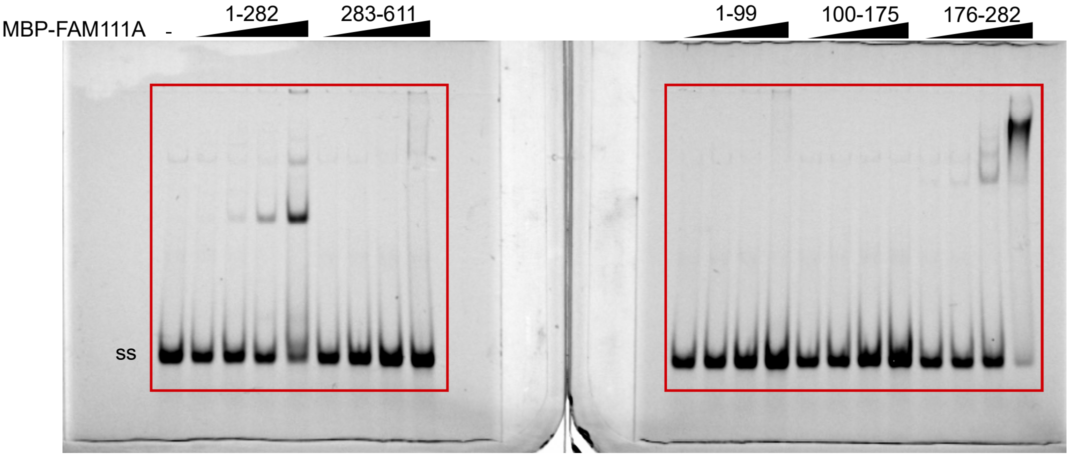

Fig. 2f

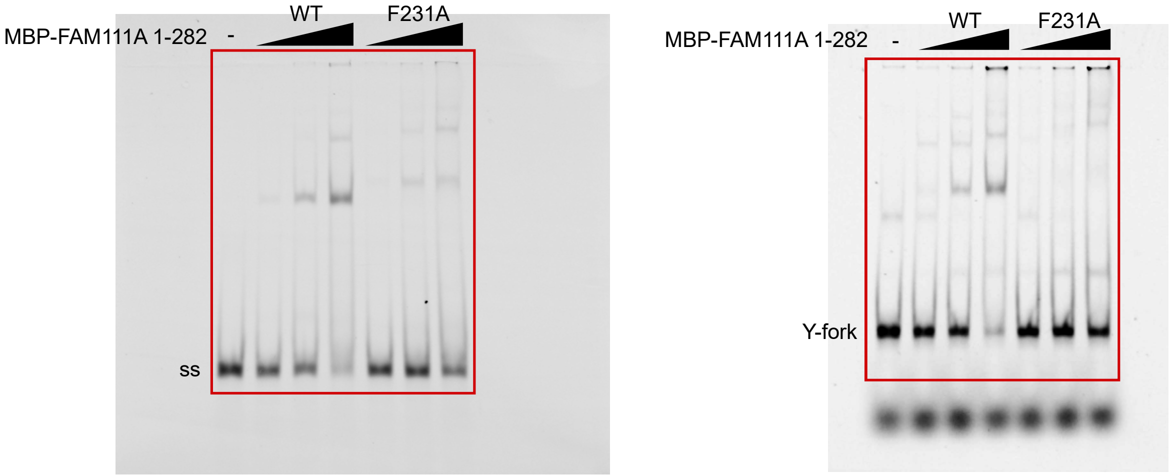

Supplementary Figure 7

Fig. 3a

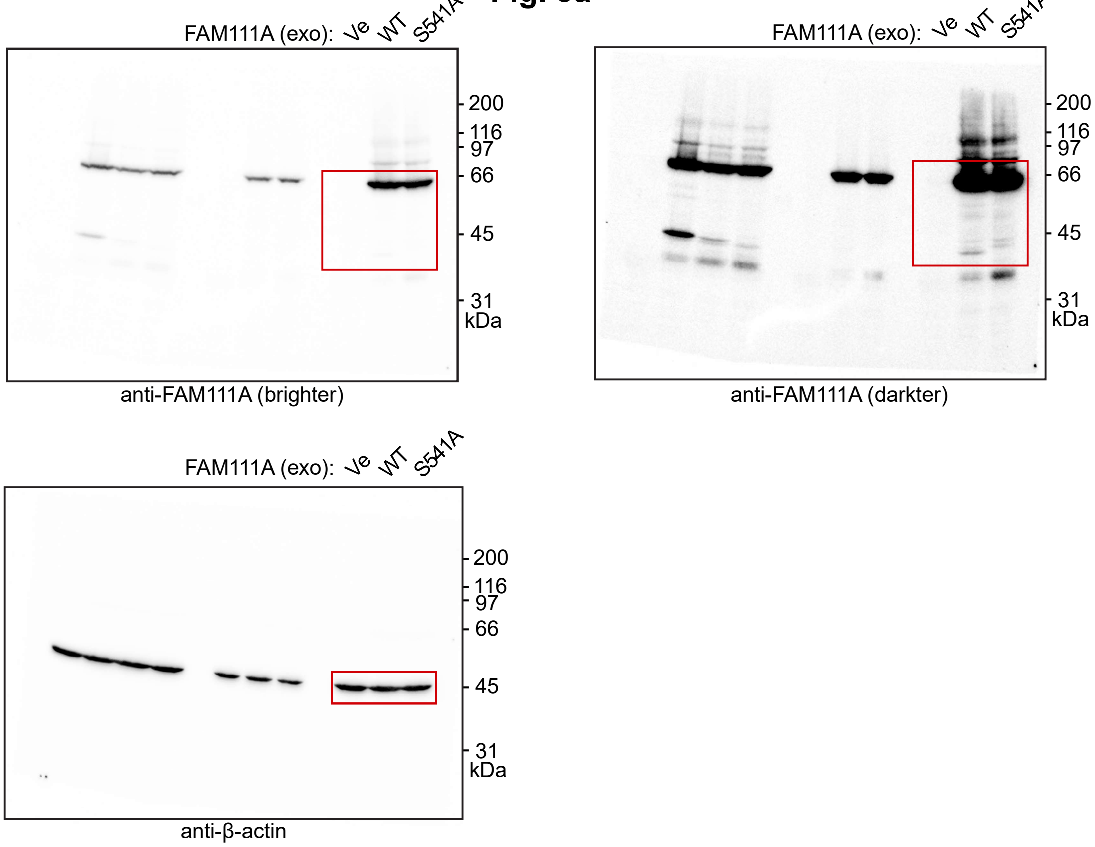

Fig. 3b

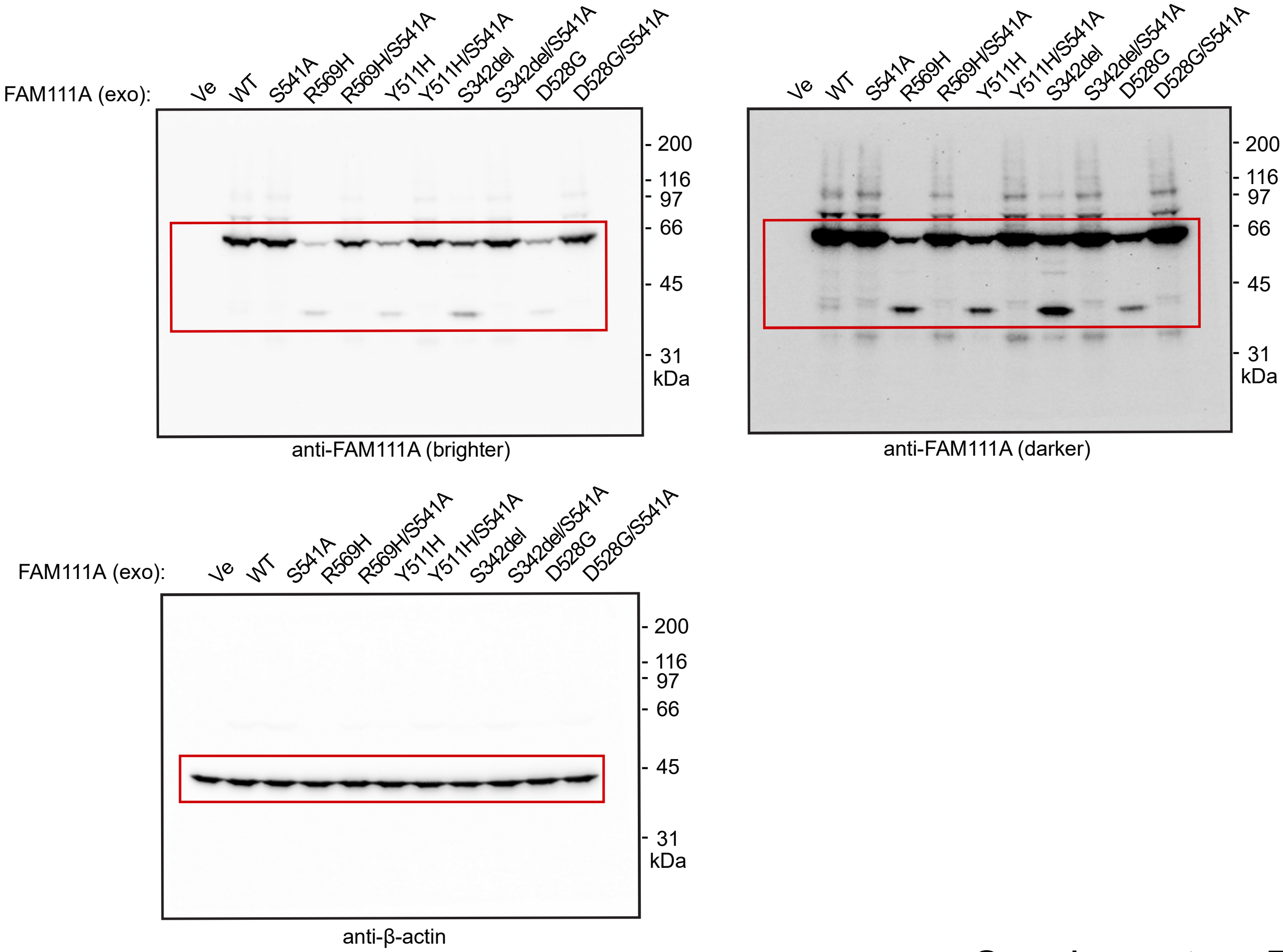

Supplementary Figure 7

Fig. 3c

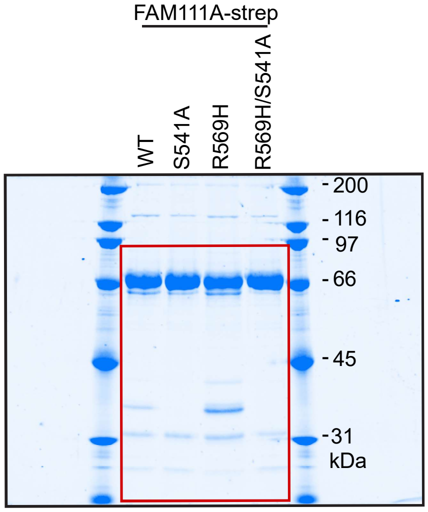

Fig. 3e

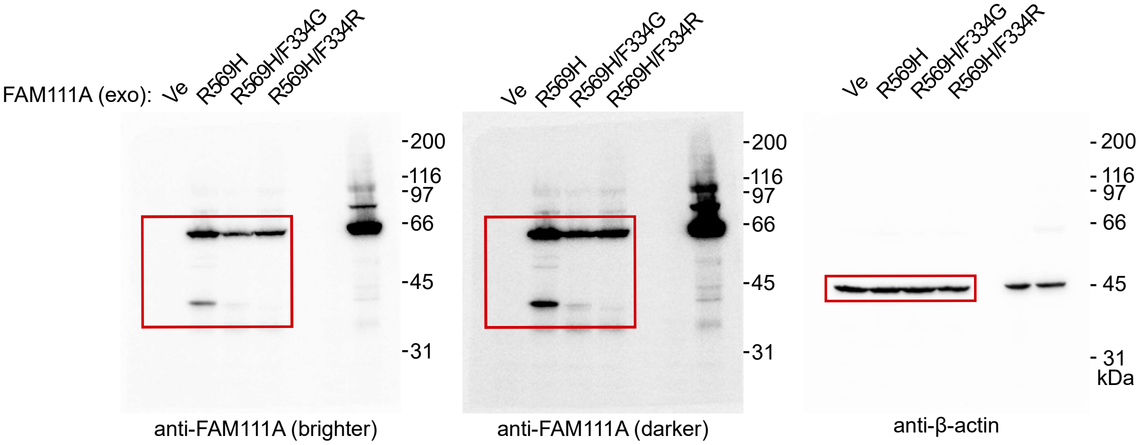

Fig. 3g

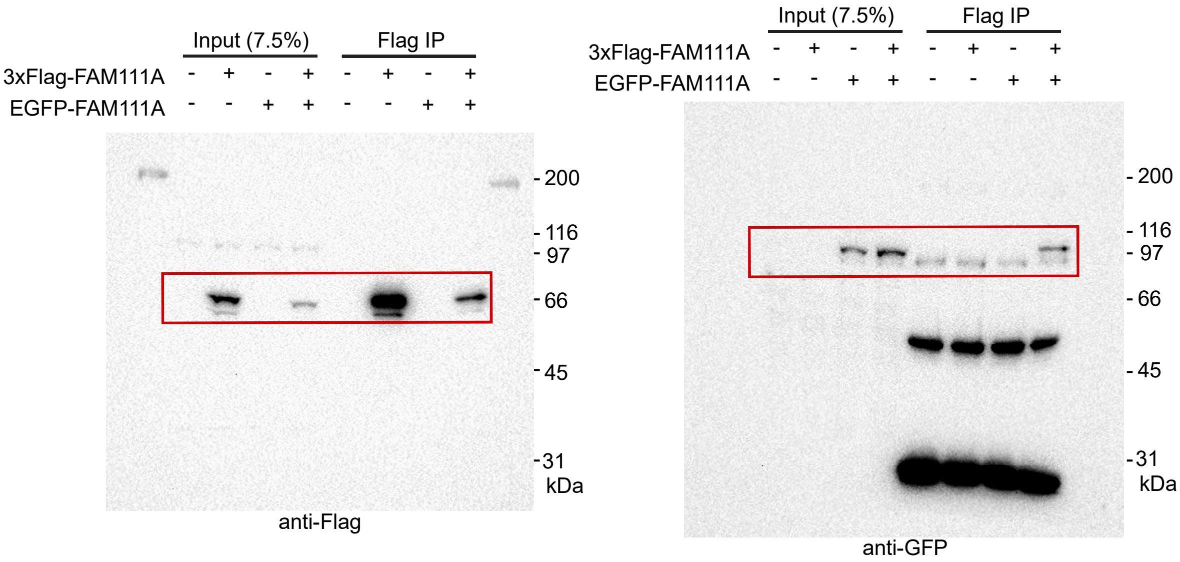

Supplementary Figure 7

Fig. 3h

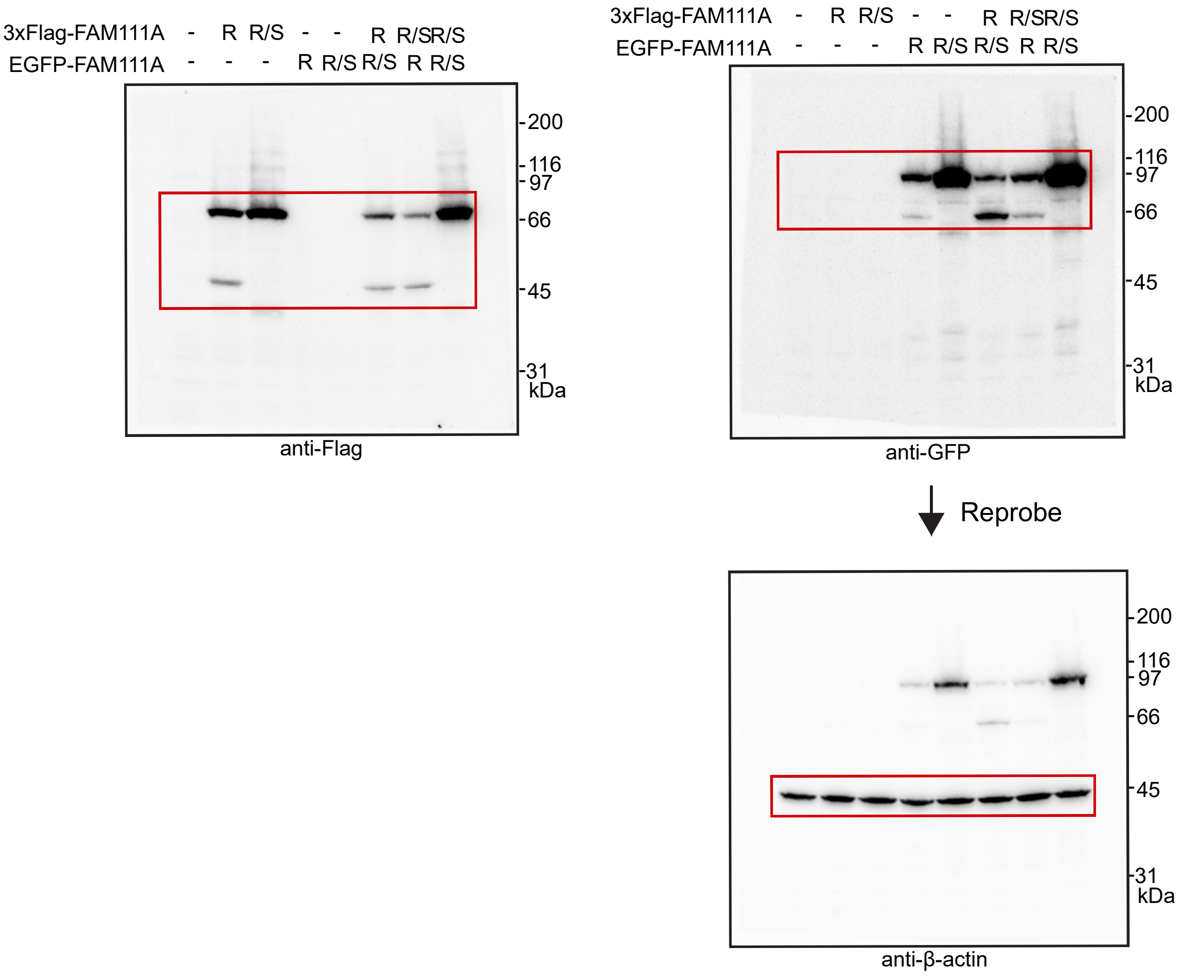

Fig. 3i

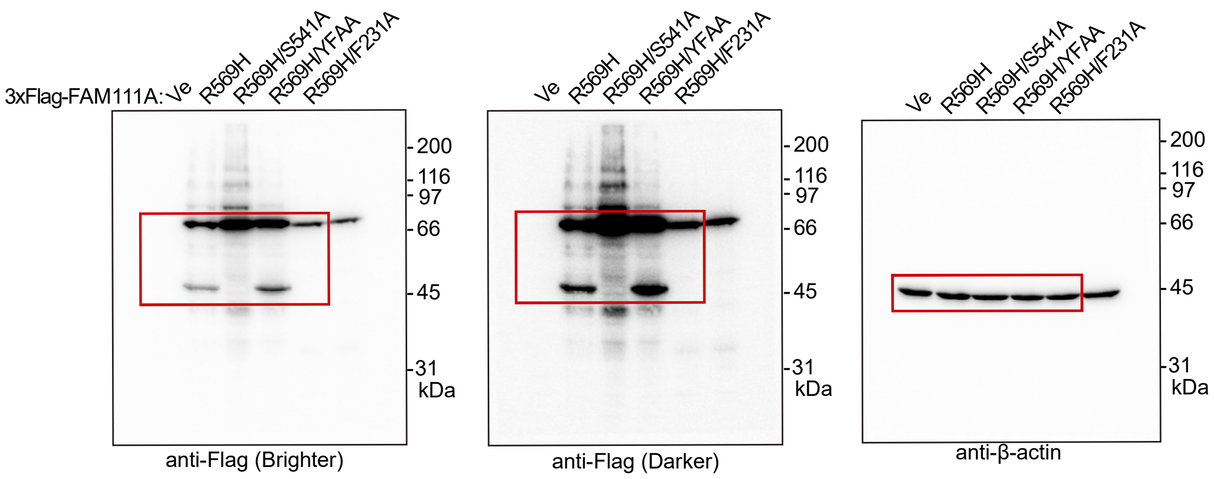

Supplementary Figure 7

Fig. 4a

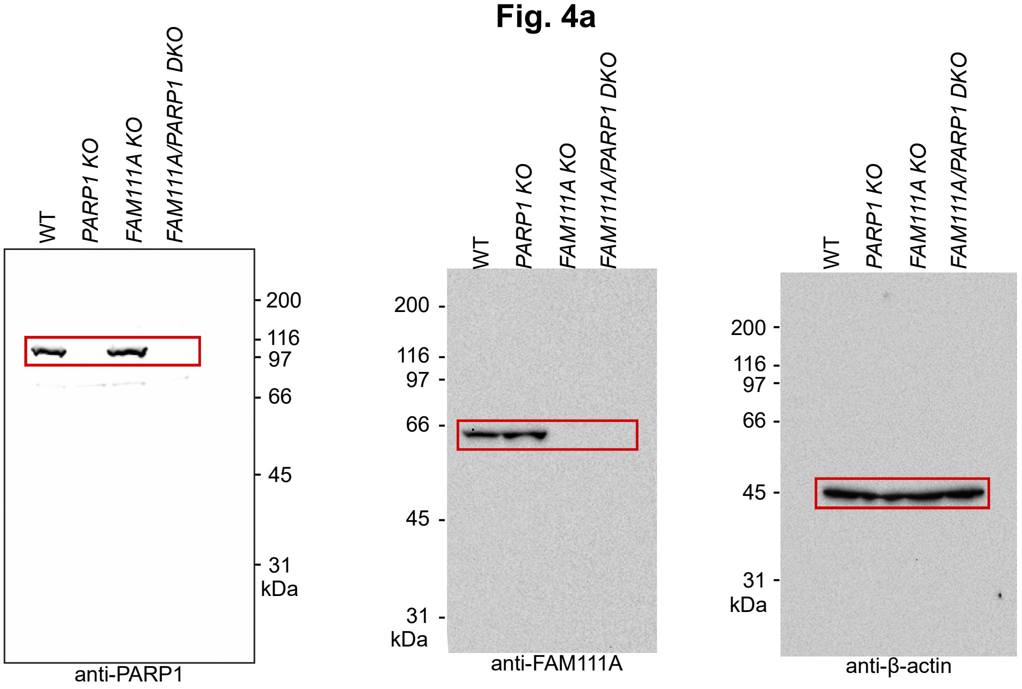

Fig. 4d

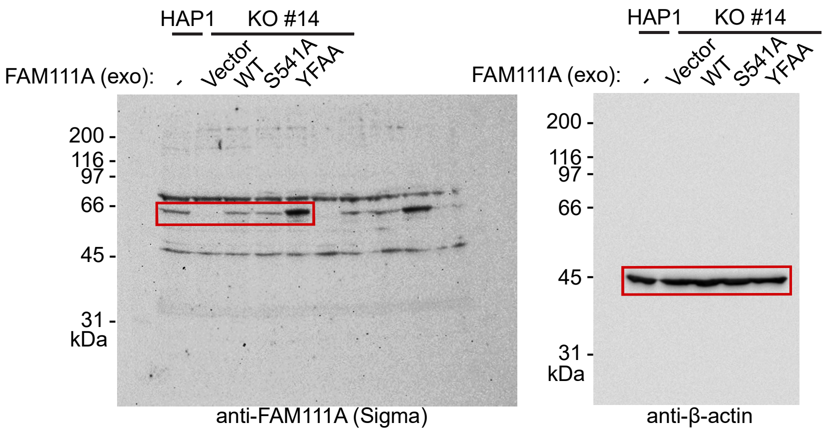

Fig. 5f

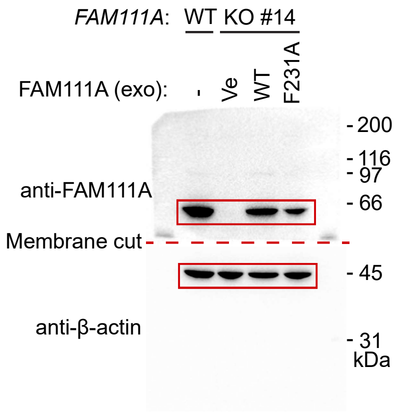

Fig. 5h

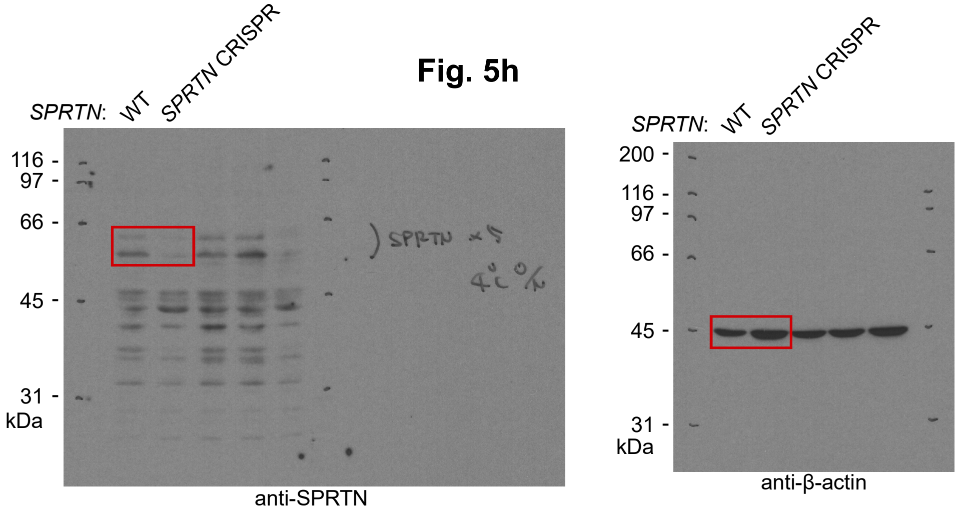

Supplementary Figure 7

Fig. 6c

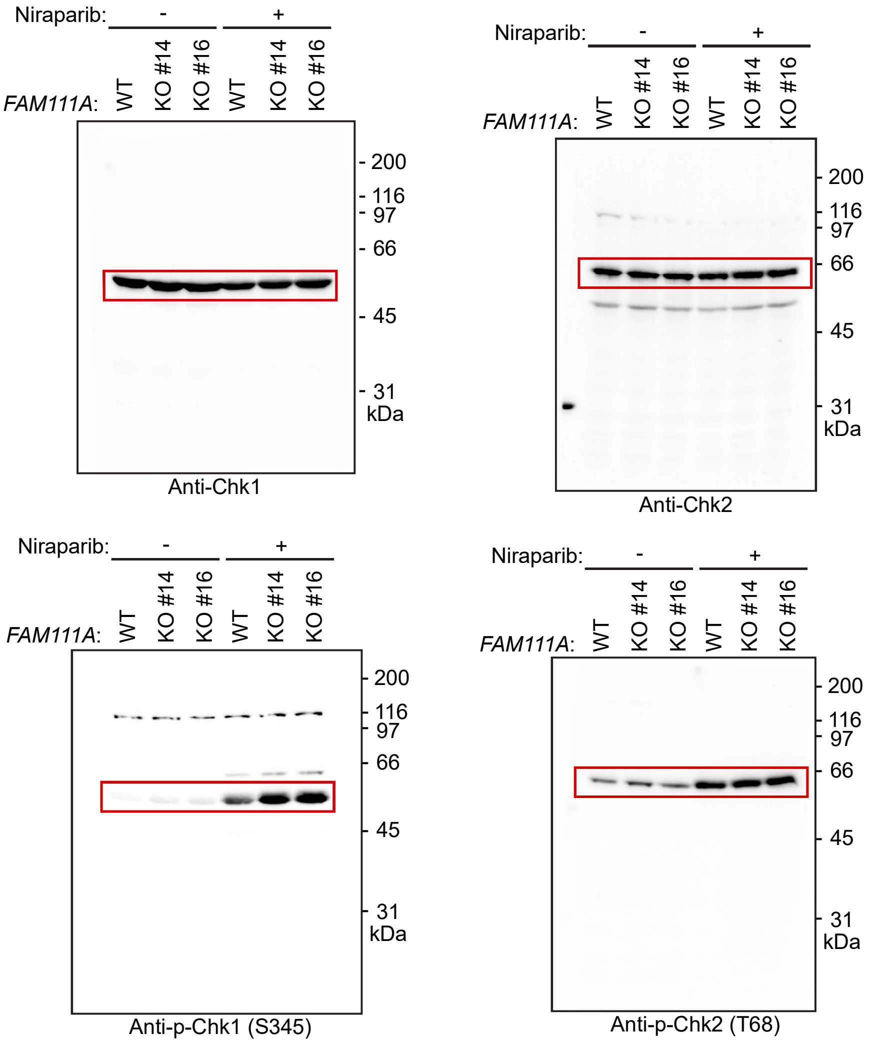

Supplementary Fig. 2a

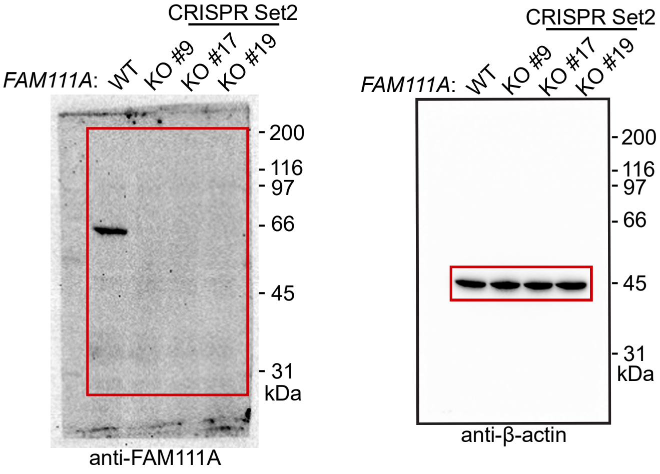

Supplementary Figure 7

Supplementary Fig. 3a

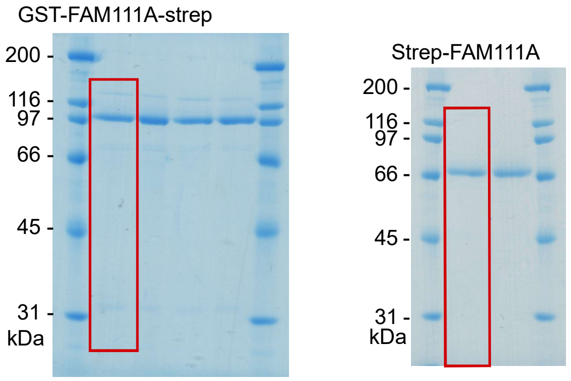

Supplementary Fig. 3b

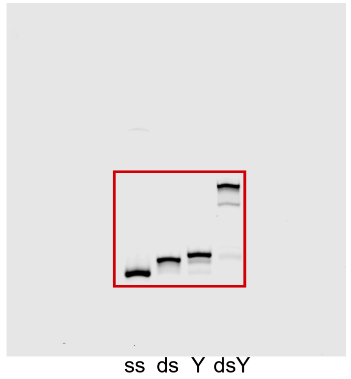

Supplementary Fig. 3c

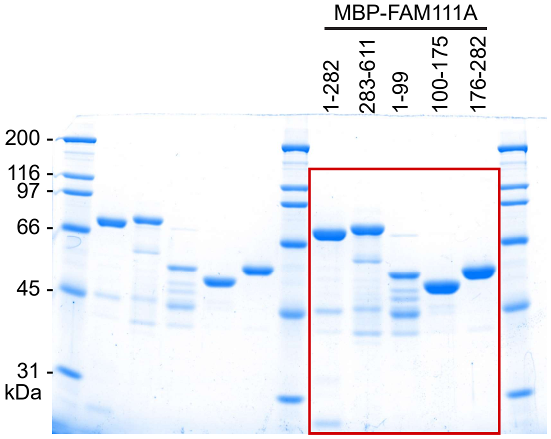

Supplementary Fig. 3e

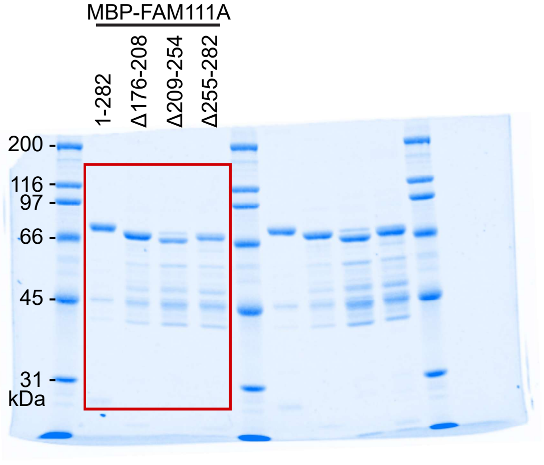

Supplementary Fig. 3f

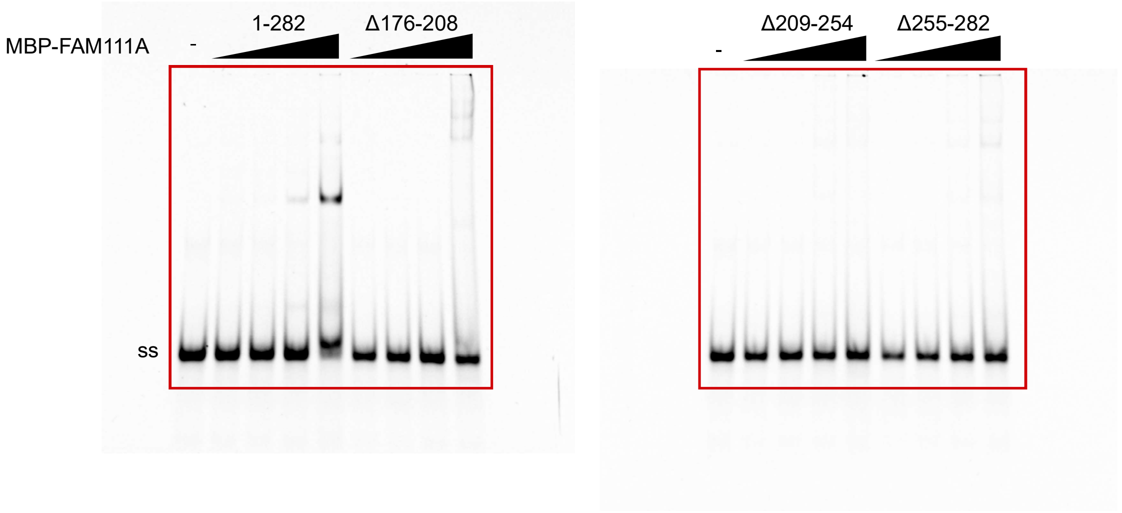

Supplementary Figure 7

Supplementary Fig. 3h

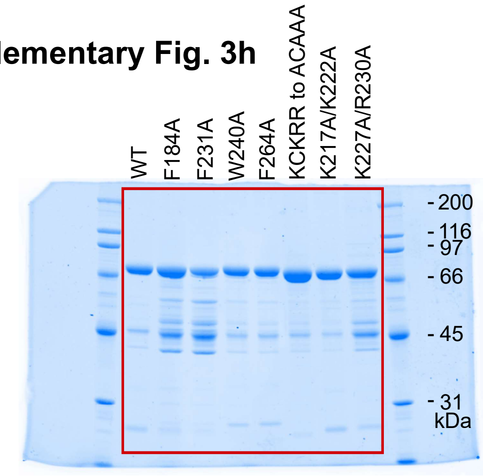

Supplementary Fig. 3i

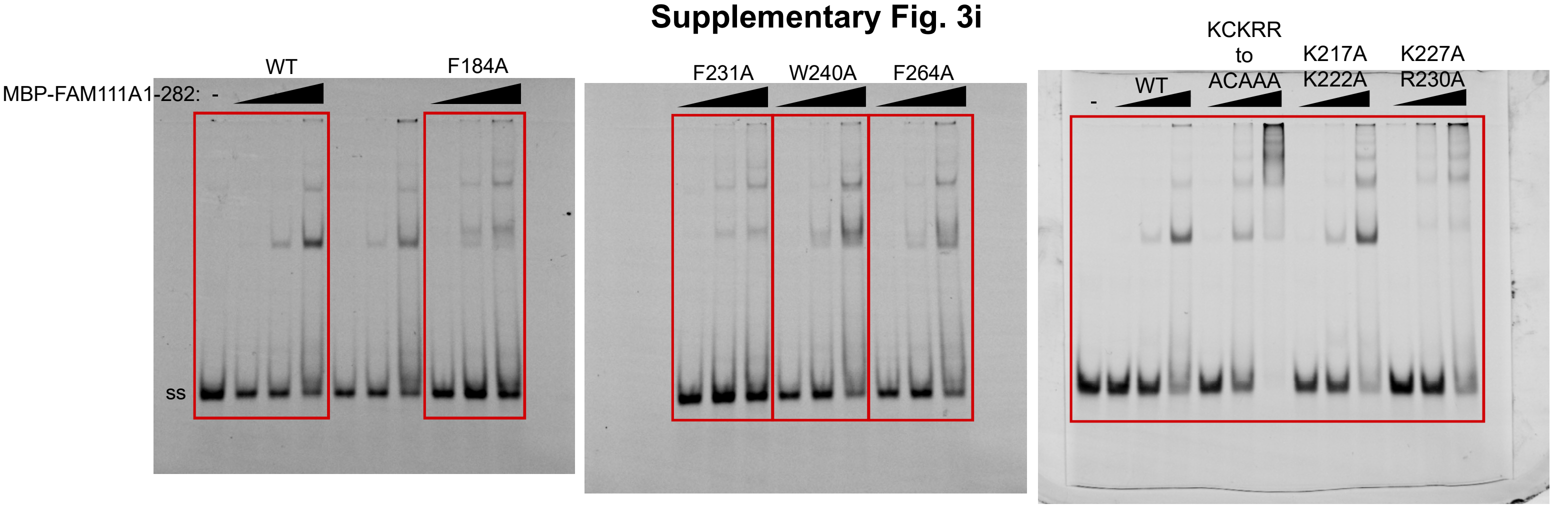

Supplementary Fig. 3j

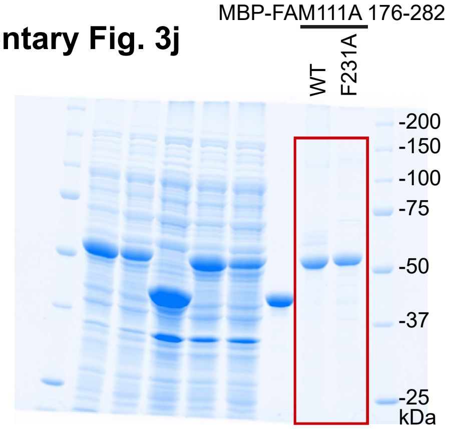

Supplementary Fig. 3k

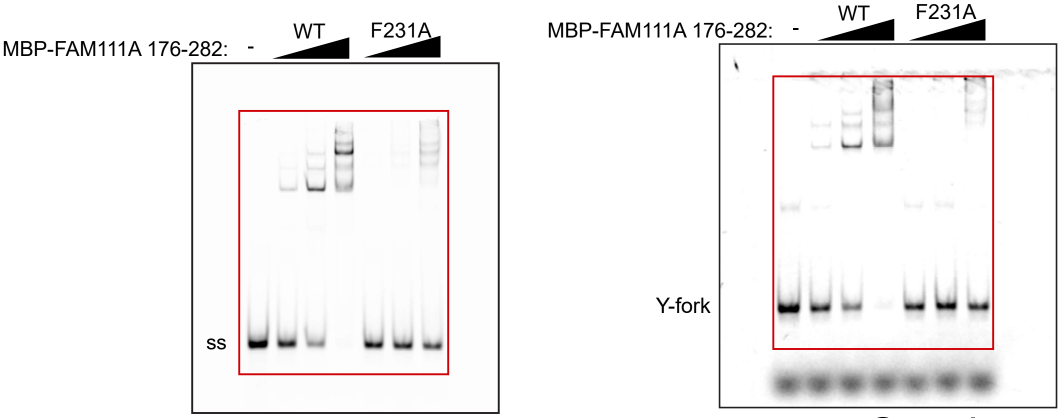

Supplementary Figure 7

Supplementary Fig. 4a

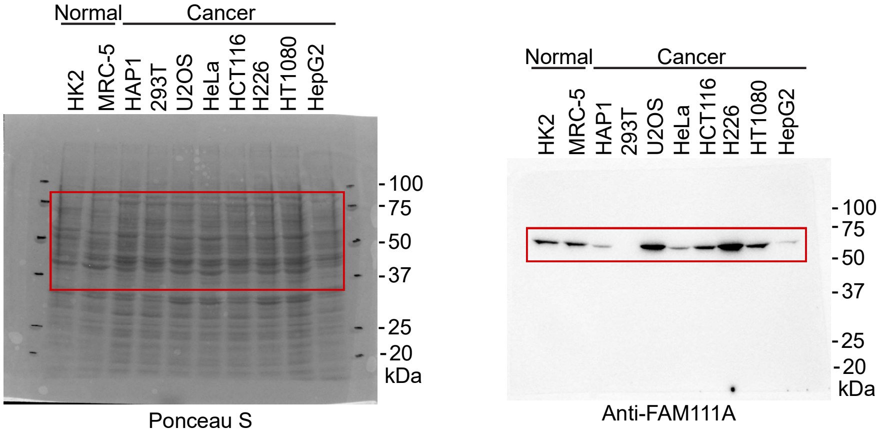

Supplementary Fig. 4c

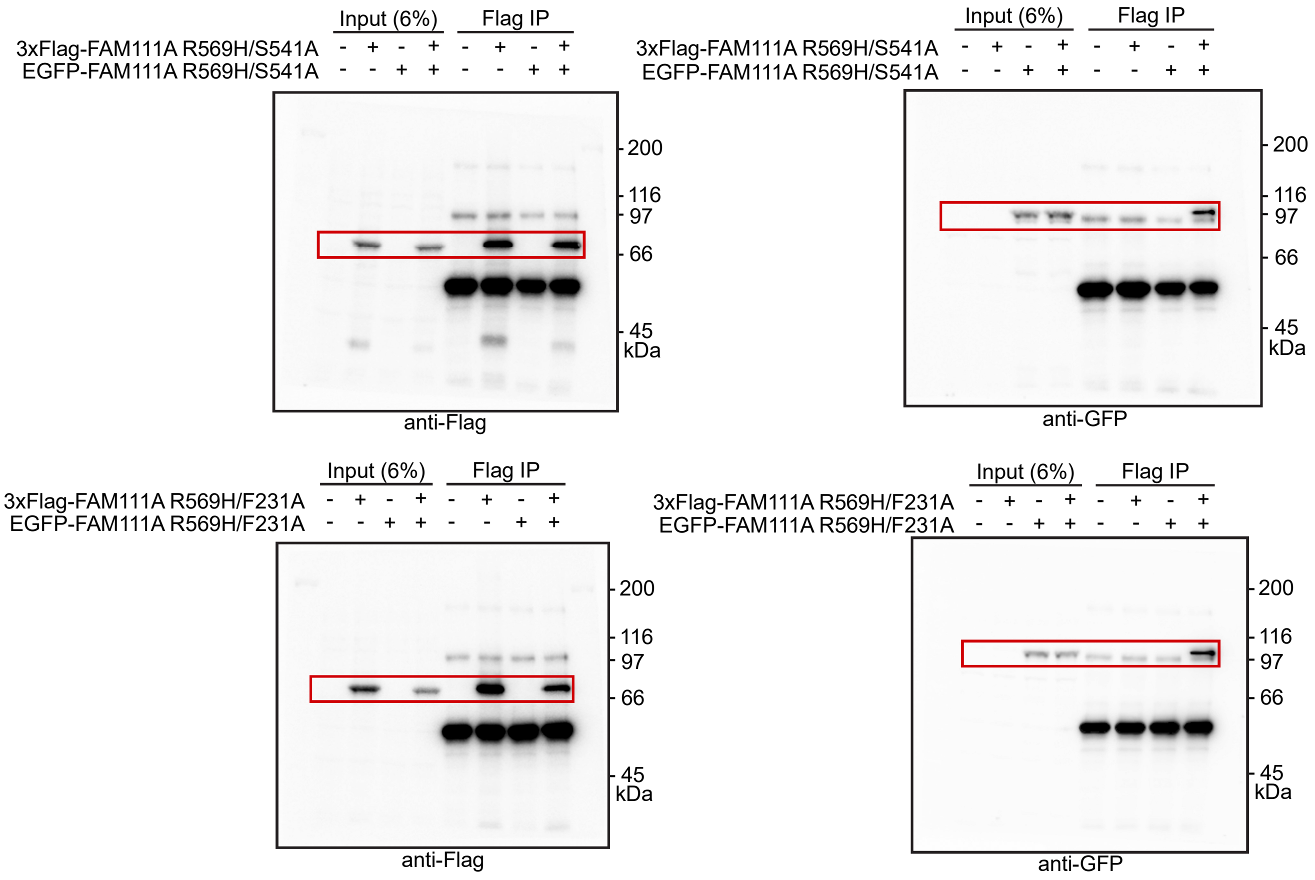

Supplementary Figure 7

Supplementary Fig. 5a

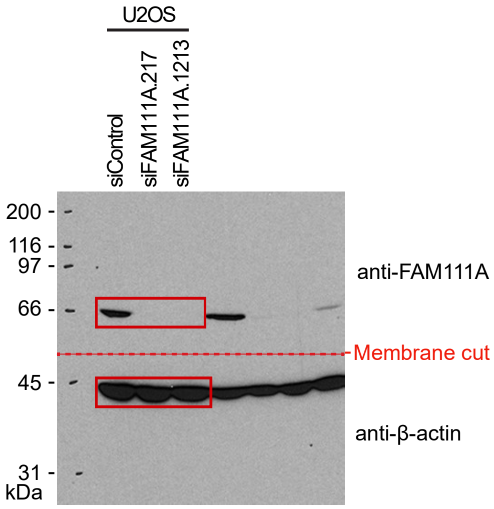

Supplementary Fig. 6a

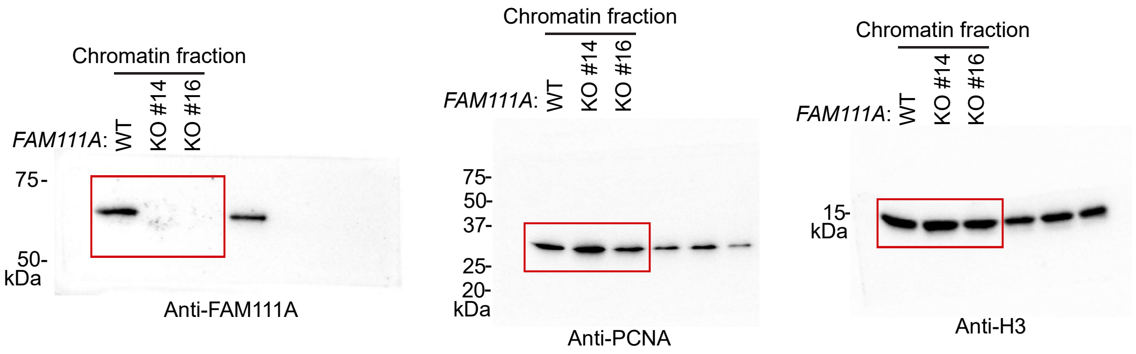

Supplementary Fig. 6d

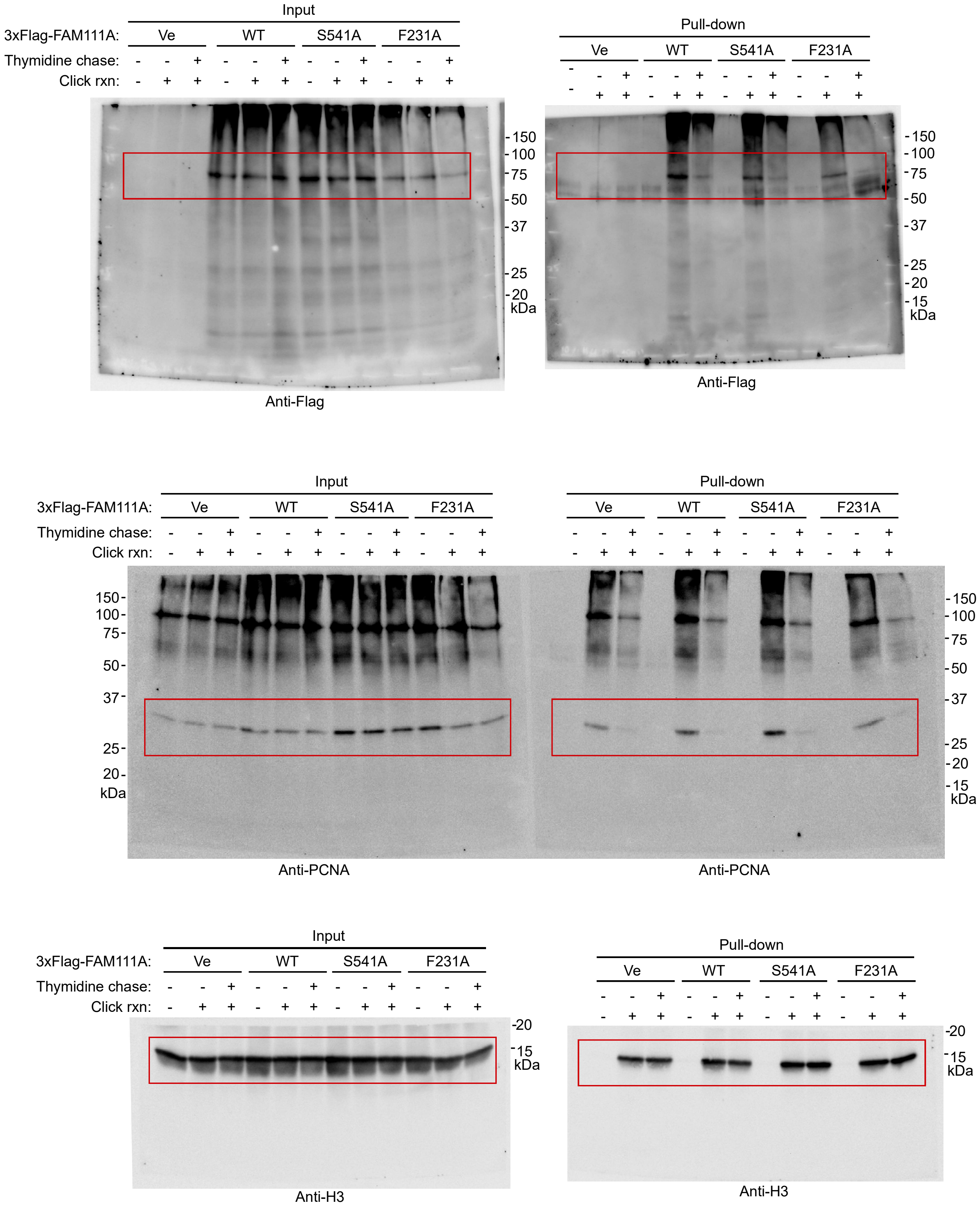

Supplementary Figure 7

Supplementary Tables

Supplementary Table 1. **Knockout cell clones used in this study.**

Target gene	Vector	gRNA	Clone Name	Mutations
FAM111A	pX335	*FAM111A* gRNA1 & 2 (Set 1)	*FAM111A* KO #14	Homozygous 45-bp insertion with a stop codon
			FAM111A KO #16	Homozygous 22-bp frameshift deletion
		FAM111A gRNA3 & 4 (Set 2)	*FAM111A* KO #9	40-bp frameshift deletion & 38-bp frameshift deletion
			FAM111A KO #17	37-bp frameshift deletion & 38-bp frameshift deletion
			FAM111A KO #19	Homozygous 37-bp frameshift deletion
PARP1	pX335	*PARP1* gRNA1 & 2	*PARP1* KO #17	Homozygous 7-bp frameshift deletion
			FAM111A/PARP1 DKO #1*	Homozygous 52-bp frameshift deletion
SPRTN	eSpCas9(1.1)	*SPRTN* gRNA5	*SPRTN* CRISPR #8	1-bp frameshift insertion & 24-bp in-frame deletion

*Derivative of *FAM111A* KO #14

Supplementary Table 2. Sequences of synthesized genes

FAM111A-strep **(codon optimized for *S. frugiperda*)**
GGGGACAAGTTTGTACAAAAAAGCAGGCTTCGCCACCATGTCCTGCAAGAAGCAGCGTTCCCGCAAGCACTCC
GTGAACGAGAAGTGCAACATGAAGATCGAGCACTACTTCAGCCCCGTGTCCAAAGAGCAGCAGAACAACTGCTC
CACCTCTCTGATGCGTATGGAATCCCGTGGCGACCCTCGTGCTACCACCAACACTCAGGCTCAGCGTTTCCACT
CTCCTAAGAAGAACCCCGAGGACCAGACCATGCCTCAGAACCGTACCATCTACGTGACCCTGAAAGTGAACCAC
CGTCGTAACCAGGACATGAAGCTGAAGCTGACCCACTCCGAGAACTCTTCCCTGTACATGGCTCTGAACACCCT
GCAGGCTGTGCGTAAAGAGATCGAGACTCACCAGGGTCAAGAGATGCTCGTGCGTGGCACCGAGGGTATCAAA
GAGTACATCAACCTGGGCATGCCTCTGTCTTGCTTCCCCGAAGGTGGACAGGTGGTCATCACCTTCAGCCAGTC
CAAGTCCAAGCAGAAAGAGGACAACCACATCTTCGGCAGGCAGGACAAGGCTTCCACCGAGTGCGTGAAGTTC
TACATCCACGCTATCGGTATCGGCAAGTGCAAGCGTCGTATCGTGAAGTGCGGCAAGCTGCACAAGAAGGGTC
GCAAGCTCTGCGTGTACGCTTTCAAGGGCGAGACTATCAAGGACGCTCTGTGCAAGGACGGTCGTTTCCTGTCC
TTCCTGGAAAACGACGACTGGAAGCTGATCGAGAACAACGACACCATCCTCGAGTCCACTCAGCCCGTGGACG
AACTGGAAGGCCGTTACTTCCAGGTCGAGGTCGAGAAGCGTATGGTGCCTTCCGCTGCTGCTTCTCAGAACCCT
GAGTCCGAGAAGAGGAACACTTGCGTGCTGCGCGAGCAGATCGTGGCTCAGTACCCATCTCTGAAGCGCGAGT
CTGAGAAGATCATCGAGAACTTCAAGAAAAAGATGAAAGTCAAGAACGGCGAGACTCTGTTCGAGCTGCACCGT
ACCACTTTCGGCAAAGTGACCAAGAACTCCTCCAGCATCAAGGTGGTCAAGCTGCTCGTCCGTCTGTCCGACTC
TGTGGGTTACCTGTTCTGGGACTCCGCTACCACCGGTTACGCTACCTGCTTCGTGTTCAAGGGCCTGTTCATCC
TGACCTGCCGTCACGTGATCGACTCCATCGTTGGCGACGGTATCGAGCCCTCTAAGTGGGCTACCATCATCGGC
CAATGCGTGCGTGTGACCTTCGGTTACGAGGAACTGAAGGACAAAGAGACTAACTACTTCTTCGTCGAGCCTTG
GTTTGAGATCCACAACGAAGAACTGGACTACGCCGTGCTGAAGCTCAAAGAAAACGGCCAGCAGGTCCCCATG
GAACTGTACAACGGTATCACCCCTGTGCCACTGTCCGGCCTGATCCACATCATCGGTCACCCTTACGGCGAGAA
GAAGCAGATCGACGCCTGCGCTGTGATCCCTCAAGGACAGCGTGCTAAGAAATGCCAAGAGCGTGTGCAGTCC
AAGAAGGCTGAGTCCCCTGAGTACGTGCACATGTACACCCAGCGCAGCTTCCAAAAGATCGTGCACAACCCCGA
CGTGATCACCTACGATACCGAGTTCTTCTTCGGTGCTTCCGGTTCTCCCGTGTTCGACTCTAAGGGTTCCCTGGT
GGCTATGCACGCTGCTGGTTTCGCTTACACCTACCAGAACGAGACTCGCTCCATCATCGAGTTCGGTTCCACCA
TGGAATCCATCCTGCTGGACATCAAGCAGCGTCACAAGCCTTGGTACGAAGAGGTGTTCGTGAACCAGCAGGAC
GTCGAGATGATGTCCGACGAGGACCTGTCCGCTTGGTCACACCCTCAGTTCGAGAAAGGTGGTGGTTCCGGTG
GCGGTTCTGGTGGTTCAGCTTGGAGTCACCCACAATTCGAGAAGTAAACCCAGCTTTCTTGTACAAAGTGGTGG
GG
FAM111A 1-282 **(codon optimized for *E. coli*)**
GGGGACAAGTTTGTACAAAAAAGCAGGCTCCGGATCCACCATGAGCTGTAAAAAACAGCGTAGCCGTAAACACA
GCGTGAATGAAAAATGCAACATGAAGATCGAGCACTATTTTAGTCCGGTTAGCAAAGAACAGCAGAATAATTGTA
GCACCAGCCTGATGCGTATGGAAAGCCGTGGTGATCCGCGTGCAACCACCAATACACAGGCACAGCGTTTTCAT
AGCCCGAAAAAAAACCCGGAAGATCAGACCATGCCGCAGAATCGTACCATTTATGTTACCCTGAAAGTGAATCAT
CGTCGCAACCAGGATATGAAACTGAAACTGACCCATAGCGAAAATAGCAGCCTGTATATGGCACTGAATACCCT
GCAGGCAGTTCGTAAAGAAATTGAAACCCATCAGGGTCAAGAAATGCTGGTTCGTGGCACCGAAGGTATTAAAG
AATATATCAATCTGGGTATGCCGCTGAGCTGTTTTCCGGAAGGTGGTCAGGTTGTTATTACCTTTAGCCAGAGCA
AAAGCAAACAGAAAGAAGATAACCATATCTTCGGTCGTCAGGATAAAGCAAGCACCGAATGTGTGAAATTCTATA
TTCATGCCATTGGCATCGGTAAATGCAAACGTCGTATTGTGAAATGCGGCAAACTGCATAAAAAAGGTCGTAAAC
TGTGCGTGTATGCGTTTAAAGGTGAAACCATTAAAGATGCCCTGTGCAAAGATGGTCGTTTTCTGAGCTTTCTGG
AAAACGATGATTGGAAACTGATCGAGAACAACGATACCATTCTGGAAAGCACCCAGCCGGTTGATGAACTGGAA
GGTCGTTATTTTCAGGTGGAAGTTGAAAAACGTATGGTTCCGAGCGCAGCAGCAAGCCAGAATCCGTAAGCGGC
CGCACACCCAGCTTTCTTGTACAAAGTGGTCCCC

Supplementary Table 3. Sequences of oligo nucleotide

Oligo Name	Sequence
**For *hFAM111A***	
FAM111A BamH1 For	5’-GCATGGATCCACCATGAGCTGTAAGAAGCAGAGG-3’
FAM111A NotI Rev	5’-GCATGCGGCCGCTCACAAGTCCTCATCACTCATC-3’
FAM111A 283aa BamHI For	5’-GGGGGGATCCGAGTCAGAGAAAAGAAACACC-3’
FAM111A S541A For	5’-CTGAATTTTTCTTTGGGGCCGCGGGCTCCCCTGTGTTTG-3’
FAM111A S541A Rev	5’-CAAACACAGGGGAGCCCGCGGCCCCAAAGAAAAATTCAG-3’
FAM111A R569H For	5’-GCTTATACTTACCAAAATGAGACTCATAGTATCATTGAGTTTGGC-3’
FAM111A R569H Rev	5’-GCCAAACTCAATGATACTATGAGTCTCATTTTGGTAAGTATAAGC-3’
FAM111A Y511H For	5’-CTAAAAAAGCAGAAAGTCCAGAGCATGTCCATATGTATACTCAAAG-3’
FAM111A Y511H Rev	5’-CTTTGAGTATACATATGGACATGCTCTGGACTTTCTGCTTTTTTAG-3’
FAM111A S342del For	5’-CGTTTGGGAAAGTAACAAAGAATTCTTCGATTAAAGTAGTGAAACTTC-3’
FAM111A S342del Rev	5’-GAAGTTTCACTACTTTAATCGAAGAATTCTTTGTTACTTTCCCAAACG-3’
FAM111A D528G For	5'-CAGAAAATAGTTCACAACCCTGGTGTGATTACCTATGACACTG-3'
FAM111A D528G Rev	5'-CAGTGTCATAGGTAATCACACCAGGGTTGTGAACTATTTTCTG-3'
FAM111A F334G For	5’-CATTATTTGAATTGCATAGAACAACGAGAGGGAAAGTAACAAAAAATT CTTCTTC-3’
FAM111A F334G Rev	5’-GAAGAAGAATTTTTTGTTACTTTCCCACCCGTTGTTCTATGCAATTCAA ATAATG-3’
FAM111A F334R For	5’-CATTATTTGAATTGCATAGAACAACGAGAGGGAAAGTAACAAAAAATT CTTCTTC-3’
FAM111A F334R Rev	5’-GAAGAAGAATTTTTTGTTACTTTCCCTCTCGTTGTTCTATGCAATTCAA ATAATG-3’
FAM111A YFAA For	5'-CACGGAAGCACTCAGTTAACGAAAAATGTAATATGAAAATCGAGCACG CTGCTTCTCCGGTCTCTAAAGAG-3’
FAM111A YFAA Rev	5’-CTCTTTAGAGACCGGAGAAGCAGCGTGCTCGATTTTCATATTACATTT TTCGTTAACTGAGTGCTTCCGTG-3’
FAM111A F231A For	5’-TGCAAGGATGGCAGAGCTCTTTCCTTTCTGGAG-3’
FAM111A F231A Rev	5’-CTCCAGAAAGGAAAGAGCTCTGCCATCCTTGCA-3’
**For codon-optimized *FAM111A (S. frugiperda)***	
N-strep-FAM111A CO Sf attB1 BamHI For	5’-GGGGACAAGTTTGTACAAAAAAGCAGGCTCCGGATCCACCATGGCT TCCGCTTGGTCACACCCTCAGTTCGAGAAAGGT-3’
N-strep-FAM111A CO Sf attB2 NotI Rev	5’-GGGGACCACTTTGTACAAGAAAGCTGGGTGTGCGGCCGCTTACAGG TCCTCGTCGGAC-3’
N-strep-FAM111A CO Sf junction For	5’-TTCGAGAAGTCCGGAATGTCCTGCAAGAAG-3’
N-strep-FAM111A CO Sf junction Rev	5’-CTTCTTGCAGGACATTCCGGACTTCTCGAATTGTGGGTGACTCCAAG CTGAACC-3’
FAM111A CO Sf S541A For	5’-GTTCTTCTTCGGTGCTGCTGGTTCTCCCGTGTTC-3’
FAM111A CO Sf S541A Rev	5’-GAACACGGGAGAACCAGCAGCACCGAAGAAGAAC-3’
FAM111A CO Sf R569H For	5’-ACCAGAACGAGACTCACTCCATCATCGAGTT-3’
FAM111A CO Sf R569H Rev	5’-AACTCGATGATGGAGTGAGTCTCGTTCTGGT-3’

Oligo name	Sequence
**For codon-optimized *FAM111A (E. coli)***	
FAM111A CO E.coli 1aa attB1 BamHI For	5'-GGGGACAAGTTTGTACAAAAAAGCAGGCTCCGGATCCACCAT GAGCTGTAAAAAACAGCGTAGCC-3'
FAM111A CO E.coli 100aa attB1 BamHI For	5'-GGGGACAAGTTTGTACAAAAAAGCAGGCTCCGGATCCACCA TGGAAAATAGCAGCCTGTATATGGCAC-3'
FAM111A CO E.coli 176aa attB1 BamHI For	5'-GGGGACAAGTTTGTACAAAAAAGCAGGCTCCGGATCCACCAT GAAAGCAAGCACCGAATGTGTGAAATTC-3'
FAM111A CO E.coli 99aa attB2 NotI Rev	5'-GGGGACCACTTTGTACAAGAAAGCTGGGTGTGCGGCCGCTT AGCTATGGGTCAGTTTCAGTTTCATATC-3'
FAM111A CO E.coli 175aa attB2 NotI Rev	5'-GGGGACCACTTTGTACAAGAAAGCTGGGTGTGCGGCCGCTT AATCCTGACGACCGAAGATATGGTTATC-3'
FAM111A CO E.coli 282aa attB2 NotI Rev	5'-GGGGACAAGTTTGTACAAAAAAGCAGGCTCCGGATCCACCA TGGAAAATAGCAGCCTGTATATGGCAC-3'
FAM111A CO E.coli Δ176-208 For	5'-TTCGGTCGTCAGGATCGTAAACTGTGCGTGTATGC-3'
FAM111A CO E.coli Δ176-208 Rev	5'-CACGCACAGTTTACGATCCTGACGACCGAAGATATGG-3'
FAM111A CO E.coli Δ209-254 For	5'-CTGCATAAAAAAGGTCCGGTTGATGAACTGGAAGG-3'
FAM111A CO E.coli Δ209-254 Rev	5'-CAGTTCATCAACCGGACCTTTTTTATGCAGTTTGCCGC-3'
FAM111A CO E.coli 254aa attB2 NotI Rev	5'-GGGGACCACTTTGTACAAGAAAGCTGGGTGTGCGGCCGCTT ACTGGGTGCTTTCCAGAATGGTATCG-3'
FAM111A CO E.coli F184A For	5'-CACCGAATGTGTGAAAGCCTATATTCATGCCATTG-3'
FAM111A CO E.coli F184A Rev	5'-CAATGGCATGAATATAGGCTTTCACACATTCGGTG-3'
FAM111A CO E.coli F231A For	5'-GTGCAAAGATGGTCGTGCTCTGAGCTTTCTGGAAAAC-3'
FAM111A CO E.coli F231A Rev	5'-GTTTTCCAGAAAGCTCAGAGCACGACCATCTTTGCAC-3'
FAM111A CO E.coli W240A For	5'-CTGGAAAACGATGATGCGAAACTGATCGAGAAC-3'
FAM111A CO E.coli W240A Rev	5'-GTTCTCGATCAGTTTCGCATCATCGTTTTCCAG-3'
FAM111A CO E.coli F264A For	5'-CTGGAAGGTCGTTATGCTCAGGTGGAAGTTGAAAAAC-3'
FAM111A CO E.coli F264A Rev	5'-GTTTTTCAACTTCCACCTGAGCATAACGACCTTCCAG-3'
FAM111A CO E.coli KCKRR to ACAAA For	5'-TGGCATCGGTGCATGCGCTGCCGCGATTGTGAAATGCGGCA AACTGC-3'
FAM111A CO E.coli KCKRR to ACAAA Rev	5'-ATTTCACAATCGCGGCAGCGCATGCACCGATGCCAATGGCATGAA-3'
FAM111A CO E.coli K217A/K222A For	5'-GTATGCGTTTGCCGGTGAAACCATTGCGGATGCCCTGTGCAA AGATGG-3'
FAM111A CO E.coli K217A/K222A Rev	5'-ACAGGGCATCCGCAATGGTTTCACCGGCAAACGCATACACG CACAGTTTAC-3'
FAM111A CO E.coli K227A/R230A For	5'-TGCCCTGTGCGCCGATGGTGCGTTTCTGAGCTTTCTGGAAAACG-3'
FAM111A CO E.coli K227A/R230A Rev	5'-AGCTCAGAAACGCACCATCGGCGCACAGGGCATCTTTAATGG-3'

Oligo name	Sequence
**For CRISPR/Cas9**	
FAM111A gRNA1 S	5’-CACCGATCAACCCACTAAGTGGCAC-3’
FAM111A gRNA1 AS	5’-AAACGTGCCACTTAGTGGGTTGATC-3’
FAM111A gRNA2 S	5’-CACCGCATATTATTGGCCATCCATA-3’
FAM111A gRNA2 AS	5’-AAACTATGGATGGCCAATAATATGC-3’
FAM111A gRNA3 S	5’-CACCGATTATGGTTGCCCACTTACT-3’
FAM111A gRNA3 AS	5’-AAACAGTAAGTGGGCAACCATAATC-3’
FAM111A gRNA4 S	5’-CACCGCAATGTGTAAGGGTGACATT-3’
FAM111A gRNA4 AS	5’-AAACAATGTCACCCTTACACATTGC-3’
PARP1 gRNA1 S	5’-CACCGCCACCTCAACGTCAGGGTGC-3’
PARP1 gRNA1 AS	5’-AAACGCACCCTGACGTTGAGGTGGC-3’
PARP1 gRNA2 S	5’-CACCGTGGGTTCTCTGAGCTTCGGT-3’
PARP1 gRNA2 AS	5’-AAACACCGAAGCTCAGAGAACCCAC-3’
SPRTN gRNA5 S	5’-CACCGAAGGGGTTCGCTGAGACGGA-3’
SPRTN gRNA5 AS	5’-AAACTCCGTCTCAGCGAACCCCTTC-3’
FAM111A Ex4 F1 (set 1 mutation check)	5’-GTAAAACGACGGCCAGTGGGTGACATTTGGTTATGAAGAGC-3’
FAM111A Ex4 R1 (set 1 mutation check)	5’-GTATAAGCAAAGCCAGCAGCATG-3’
FAM111A Ex4 F2 (set 2 mutation check)	5'-ACAGTGCAACTACGGGTTACG-3'
FAM111A Ex4 R2 (set 2 mutation check)	5'-TTCAGGACAGCATAGTCAAGC-3'
PARP1 Ex2 F (mutation check)	5'-CAAGGTCAAGGTCTAGTGGGTC-3'
PARP1 Ex2 R (mutation check)	5'-ACCATACGCTTGATCTGCAC-3'
SPRTN Ex2 F2 (mutation check)	5'-AAGAATGTGTAAGGACTTGGGC-3'
SPRTN Ex2 R1 (mutation check)	5'-CACAGCAGTCTAAATCATTGACAAG-3'
**For EMSA and FP assay**	
IRDye700-O3063	5’- (IRDye700)-GTTTTCCCAGTCACGACGATGCTCCGGTACTCCA GTGTAGGCATATTACGAATTCTTGAGGCAGGCATGGTAGCT-3’
O4786	5’-AGCTACCATGCCTGCCTCAAGAATTCGTAATATGCCTACACT GGAGTACCGGAGCATCGTCGTGACTGGGAAAAC-3’
O4787	5’-AGCTACCATGCCTGCCTCAAGAATTCGTAATATGCCTACACT GGACCGTACTTCGCCTAGTAGACTGCCTTCCCG-3’
O3730	5’-GTACCGGAGCATCGTCGTGACTGGGAAAAC-3’
O3757	5’-CGGGAAGGCAGTCTACTAGGCGAAGTACGG-3’
6-FAM-O1676	5’- (6-FAM)-CGACGATGCTCCGGTACTCCAGTGTAGGCAT-3’
**siRNA**	
siControl S	5’-CGUACGCGGAAUACUUCGA-dTdT-3’
siControl AS	5’-UCGAAGUAUUCCGCGUACG-dTdT-3’
siFAM111A.271 S	5’-CUAAAGAGCAACAGAAUAA-dTdT-3’
siFAM111A.271 AS	5’-UUAUUCUGUUGCUCUUUAG-dTdT-3’
siFAM111A.1213 S	5’-CGAUUAAAGUAGUGAAACU-dTdT-3’
siFAM111A.1213 AS	5’-AGUUUCACUACUUUAAUCG-dTdT-3’

Supplementary Table 3
