# Supplementary material for: FAM111A protects replication forks from protein obstacles via its trypsin-like domain
Source: Nat Commun. 2020 Mar 12;11:1318. doi: 10.1038/s41467-020-15170-7 (PMC7067828; doi:10.1038/s41467-020-15170-7)
Supplement: Supplementary file 3 — Reporting Summary [file 41467_2020_15170_MOESM3_ESM.pdf]

nature research

Corresponding author(s): Yuichi J. Machida

Last updated by author(s): Jan 31, 2020

Reporting Summary

Nature Research wishes to improve the reproducibility of the work that we publish. This form provides structure for consistency and transparency in reporting. For further information on Nature Research policies, see Authors & Referees and the Editorial Policy Checklist.

Statistics

For all statistical analyses, confirm that the following items are present in the figure legend, table legend, main text, or Methods section.

n/a	Confirmed	
☐	☒	The exact sample size (*n*) for each experimental group/condition, given as a discrete number and unit of measurement
☐	☒	A statement on whether measurements were taken from distinct samples or whether the same sample was measured repeatedly
☐	☒	The statistical test(s) used AND whether they are one- or two-sided<br>*Only common tests should be described solely by name; describe more complex techniques in the Methods section.*
☒	☐	A description of all covariates tested
☒	☐	A description of any assumptions or corrections, such as tests of normality and adjustment for multiple comparisons
☐	☒	A full description of the statistical parameters including central tendency (e.g. means) or other basic estimates (e.g. regression coefficient) AND variation (e.g. standard deviation) or associated estimates of uncertainty (e.g. confidence intervals)
☒	☐	For null hypothesis testing, the test statistic (e.g. *F*, *t*, *r*) with confidence intervals, effect sizes, degrees of freedom and *P* value noted<br>*Give P values as exact values whenever suitable.*
☒	☐	For Bayesian analysis, information on the choice of priors and Markov chain Monte Carlo settings
☒	☐	For hierarchical and complex designs, identification of the appropriate level for tests and full reporting of outcomes
☒	☐	Estimates of effect sizes (e.g. Cohen's *d*, Pearson's *r*), indicating how they were calculated

Our web collection on statistics for biologists contains articles on many of the points above.

Software and code

Policy information about availability of computer code

Data collection	ImageJ was used to score γH2AX foci. Image Studio Lite (LI-COR) and Image Lab (Bio-Rad) were used to quantify band intensities in EMSA.
Data analysis	Prism 5 (GraphPad) was used for making graphs and statistical analyses.

For manuscripts utilizing custom algorithms or software that are central to the research but not yet described in published literature, software must be made available to editors/reviewers. We strongly encourage code deposition in a community repository (e.g. GitHub). See the Nature Research guidelines for submitting code & software for further information.

Data

Policy information about availability of data

All manuscripts must include a data availability statement. This statement should provide the following information, where applicable:

- Accession codes, unique identifiers, or web links for publicly available datasets
- A list of figures that have associated raw data
- A description of any restrictions on data availability

All data supporting this study are available from the corresponding author on reasonable request.

Field-specific reporting

Please select the one below that is the best fit for your research. If you are not sure, read the appropriate sections before making your selection.

☒ Life sciences ☐ Behavioural & social sciences ☐ Ecological, evolutionary & environmental sciences

For a reference copy of the document with all sections, see nature.com/documents/nr-reporting-summary-flat.pdf

Life sciences study design

All studies must disclose on these points even when the disclosure is negative.

Sample size	n/a
Data exclusions	No data was excluded in this study.
Replication	Experiments were repeated biologically at least twice and similar results were obtained. The number of replication for each experiment was described in figure legends.
Randomization	n/a
Blinding	For focus formation and DNA combing assays, the investigator was blinded to sample identity.

Reporting for specific materials, systems and methods

We require information from authors about some types of materials, experimental systems and methods used in many studies. Here, indicate whether each material, system or method listed is relevant to your study. If you are not sure if a list item applies to your research, read the appropriate section before selecting a response.

Materials & experimental systems

n/a	Involved in the study	
☐	☒	Antibodies
☐	☒	Eukaryotic cell lines
☒	☐	Palaeontology
☒	☐	Animals and other organisms
☒	☐	Human research participants
☒	☐	Clinical data

Methods

n/a	Involved in the study	
☒	☐	ChIP-seq
☐	☒	Flow cytometry
☒	☐	MRI-based neuroimaging

Antibodies

Antibodies used	All antibodies used in this study are listed in the Methods section.
Validation	The commercial antibodies were validated by manufactures. Mouse anti-TOP1cc and anti-SPRTN antibodies have been validated in our previous publications. Mouse anti-PARP1 antibody was a kind gift from Guy Poirier and validated in this study by PARP1 KO cells.

Eukaryotic cell lines

Policy information about cell lines

Cell line source(s)	HAP1 cell line was purchased from Horizon Discovery. 293T, U2OS, HK2, MRC-5, HeLa, HCT116, H226, HT1080 and HepG2 cell lines were obtained from American Type Culture Collection (ATCC).
Authentication	Cells were authenticated by suppliers.
Mycoplasma contamination	HAP1, 293T and U2OS cell lines were tested for mycoplasma contamination by LookOut Mycoplasma PCR Detection Kit (Sigma). Other cell lines were not tested.
Commonly misidentified lines (See ICLAC register)	No commonly misidentified cell line was used.

Flow Cytometry

Plots

Confirm that:

☒ The axis labels state the marker and fluorochrome used (e.g. CD4-FITC).

☒ The axis scales are clearly visible. Include numbers along axes only for bottom left plot of group (a 'group' is an analysis of identical markers).

☒ All plots are contour plots with outliers or pseudocolor plots.

☒ A numerical value for number of cells or percentage (with statistics) is provided.

Methodology

Sample preparation	Detailed sample preparation is described in the Methods section.
Instrument	FACS Canto II (BD Bioscience)
Software	FlowJo 8.8.7 (BD), ModFit LT (Verity Software House).
Cell population abundance	Ten thousand cells were randomly analyzed from whole population for Annexin V/PI staining or from single cell population for cell cycle analyses.
Gating strategy	For cell cycle analyses, a single cell population was gated based on FSC/SSC. Estimation of cell cycle phases was performed using ModFit LT software.<br>For Annexin V/PI staining, gating was defined by control samples (with/without staining, with/without induction of cell death). Representative gating is shown in Fig. 1c.

☐ Tick this box to confirm that a figure exemplifying the gating strategy is provided in the Supplementary Information.
